# All-cause and cause-specific mortality in people with mental disorders and intellectual disabilities, before and during the COVID-19 pandemic: cohort study

**DOI:** 10.1016/j.lanepe.2021.100228

**Published:** 2021-10-07

**Authors:** Jayati Das-Munshi, Chin Kuo Chang, Ioannis Bakolis, Matthew Broadbent, Alex Dregan, Matthew Hotopf, Craig Morgan, Robert Stewart

**Affiliations:** a King's College London, Department of Psychological Medicine, Institute of Psychiatry, Psychology & Neurosciences, London, United Kingdom; b South London & Maudsley NHS Foundation Trust, London, United Kingdom; c Institute of Epidemiology and Preventive Medicine, College of Public Health, National Taiwan University, Taipei, Taiwan; d Global Health Program, College of Public Health, National Taiwan University, Taipei, Taiwan; e ESRC Centre for Society and Mental Health, King's College London, London, United Kingdom; f King's College London, Centre for Implementation Science, Health Services and Population Research Department, Institute of Psychiatry, Psychology and Neuroscience, London, United Kingdom; g King's College London, Department of Biostatistics and Health Informatics, Institute of Psychiatry, Psychology and Neuroscience, London, United Kingdom; h King's College London, Health Services and Population Research Department, Institute of Psychiatry, Psychology and Neuroscience, London, United Kingdom

## Abstract

**BACKGROUND:**

People with mental disorders and intellectual disabilities experience excess mortality compared with the general population. The impact of COVID-19 on exacerbating this, and in widening ethnic inequalities, is unclear.

**METHODS:**

Prospective data (N=167,122) from a large mental healthcare provider in London, UK, with deaths from 2019 to 2020, used to assess age- and gender-standardised mortality ratios (SMRs) across nine psychiatric conditions (schizophrenia-spectrum disorders, affective disorders, somatoform/ neurotic disorders, personality disorders, learning disabilities, eating disorders, substance use disorders, pervasive developmental disorders, dementia) and by ethnicity.

**FINDINGS:**

Prior to the World Health Organization (WHO) declaring COVID-19 a public health emergency on 30th January 2020, all-cause SMRs across all psychiatric cohorts were more than double the general population. By the second quarter of 2020, when the UK experienced substantial peaks in COVID-19 deaths, all-cause SMRs increased further, with COVID-19 SMRs elevated across all conditions (notably: learning disabilities: SMR: 9.24 (95% CI: 5.98-13.64), pervasive developmental disorders: 5.01 (95% CI: 2.40-9.20), eating disorders: 4.81 (95% CI: 1.56-11.22), schizophrenia-spectrum disorders: 3.26 (95% CI: 2.55-4.10), dementia: 3.82 (95% CI: 3.42, 4.25) personality disorders 4.58 (95% CI: 3.09-6.53)). Deaths from other causes remained at least double the population average over the whole year. Increased SMRs were similar across ethnic groups.

**INTERPRETATION:**

People with mental disorders and intellectual disabilities were at a greater risk of deaths relative to the general population before, during and after the first peak of COVID-19 deaths, with similar risks by ethnicity. Mortality from non-COVID-19/ other causes was elevated before/ during the pandemic, with higher COVID-19 mortality during the pandemic.

**FUNDING:**

ESRC (JD, CM), NIHR (JD, RS, MH), Health Foundation (JD), GSK, Janssen, Takeda (RS).

**Research in context**

**Evidence before this study**

On 30th January 2020, following the identification of novel SARS-COV2 coronavirus, the World Health Organization (WHO) declared a public health emergency of international concern (PHEIC). The first known case of COVID-19 in the UK entered the country on 23^rd^ January 2020, however since then COVID-19 has impacted on every single region in the world. People living with severe mental disorders were known to experience 15 to 20-year reductions in life expectancy prior to the pandemic, mostly from preventable physical causes. Concerns have been raised that the pandemic may have further adversely impacted on this, and there are concerns that marginalised groups with protected characteristics (e.g. ethnicity) have also been severely impacted, yet to date studies quantifying this are scarce.

We searched Medline (OVID) for population-based studies from inception until February 26^th^ 2021, using the following search terms: “mental disorder*”, “psychiatric disorder*”, “substance-related disorder*”, “mortality”, “mortality, premature” “coronavirus” and “COVID-19”. Papers were selected for inclusion if a suitable comparison control group was presented and adjustments for age and sex had been performed as a minimum, in assessments of mortality. No restrictions were placed on language. We extracted data relating to country, psychiatric diagnosis, ethnicity and deaths.

Of 132 identified papers 6 met criteria for inclusion and were from the USA (2), UK (2), Denmark (1) and South Korea (1). Of these studies, diagnostic groups assessed included schizophrenia-spectrum disorders (5), affective disorders (5), neurotic/ stress-related somatoform disorders (3), dementia (3) and substance use disorders (3). We did not find any peer-reviewed studies assessing impacts on people with personality disorders, eating disorders or learning disabilities. In three of these studies, psychiatric diagnostic groups were combined into an ‘any mental disorder’ diagnosis, for analyses. In four studies race/ ethnicity was assessed, with comparisons presented. In a cohort study from the US, compared to a control group without psychiatric disorders, adjusted hazard ratios (aHRs) (adjusted for age, sex and race) for mortality up to 45 days after a positive COVID-19 test, were substantially elevated in people with schizophrenia-spectrum disorders (aHR: 2.87 (95%CI: 1.62-5.08), less elevated in people with affective disorders (aHR: 1.25 (95% CI: 0.98-1.61), and similar in anxiety disorders (aHR: 0.97 (95% CI: 0.67-1.41). This study did not find any differences in risk of death by race/ ethnicity in the sample overall with ‘any psychiatric diagnosis’. One other US study also indicated an increased risk of death (aHR: 1.5 (95% CI 1.1-1.9)) in people with ‘any psychiatric diagnosis’ (which included all ICD-10 mental disorders, dementia and self-harm). There was no evidence in this study of differences in mortality risk by race/ ethnicity. In one study from the UK which assessed people with severe mental illnesses (including diagnoses of schizophrenia-spectrum, bipolar, and severe depression), relative to the general population, age- and sex-standardised mortality ratios showed a substantial increase over the pandemic period compared with pre-pandemic years, which was not accounted for by ethnicity. A second UK study of people with ‘any pre-pandemic mental disorder’ indicated a higher adjusted odds ratio for COVID-19 mortality compared to those without pre-pandemic mental disorders, with a similar magnitude of association noted by ethnicity. In a nation-wide cohort from Denmark, age- and sex-adjusted odds ratios (ORs) for deaths within 30 days of a positive COVID-19 PCR test were elevated in people with dementia (OR 2.0 (95% CI: 1.5-2.6)), major psychiatric disorders (OR: 2.5 (95% CI: 1.2-5.1)), alcohol abuse (OR: 1.8 (95% CI:1.2-2.7)), substance abuse (OR: 1.1 (95% CI: 1.1-3.2); the reference for these assessments were people without the indicated condition who had also had a positive SARS COV2 PCR test. In a study from South Korea of people aged 65 years or more, in people with ‘any psychiatric diagnosis’ (which included dementia and functional diagnoses such as depression, psychosis as well as substance use disorders) the hazard ratio for death was 1.57 (95% CI: 0.95-2.56), in models adjusting for age, sex and month of diagnosis, through propensity score matching.

**Added value of this study**

Our study builds on this evidence by assessing mortality risk prior to and during the COVID-19 pandemic over a range of mental disorder and intellectual disability diagnoses, and includes diagnoses (personality disorders, eating disorders and learning disabilities) for which there have been no studies with near-complete ascertainment of mortality and/or stratification by ethnicity and/or a follow up period spanning to the end of the year in which the COVID-19 pandemic was first declared. Our study confirms that people living with a range of mental disorder and intellectual disability diagnoses were already at a higher risk of all-cause mortality prior to the pandemic, compared with the general population. The COVID-19 pandemic in the UK was associated with a further steep increase in mortality risk relative to the general population, which was substantially elevated compared to the previous/ pre-pandemic period. Deaths from COVID-19, reflected in age and gender standardised mortality ratios (SMRs), were elevated across all conditions surveyed relative to the general population, this is noteworthy as deaths in the general population were already markedly elevated during the observation period. In addition, deaths from all other/ non-COVID-19 causes continued to be experienced in excess, relative to the general population, over the pandemic period across all mental disorder and intellectual disability diagnoses in the study. Our analyses suggest that after the first wave of COVID-19 infection and deaths in the UK, all-cause mortality risk reduced from a large peak but continued to remain elevated in people with mental disorder and intellectual disability diagnoses. Our study supports the view that the pandemic has widened pre-existing inequalities impacting people with mental disorders and intellectual disabilities, with similar trends noted in White British and UK ethnic minority groups living with these conditions. Our analyses suggest a similar and consistent widening of inequalities related to mortality across all conditions surveyed in this study, with an increased risk most notable for people with learning disabilities, but also observed across schizophrenia, dementia, affective disorders (including bipolar disorder and major depression), anxiety disorders, personality disorders and substance use disorders.

**Implications of all the available evidence**

The findings lend further support to concerns that people with mental disorders and intellectual disabilities are at an increased risk of death, which may be associated with COVID-19 infection and/or, potentially, policies and other changes impacting healthcare delivery which may have exacerbated inequalities during the first wave in the UK. To ensure parity of esteem between mental health and physical health, international vaccine prioritisation exercises may wish to consider mental health conditions as high priority and approaches to enhance vaccine implementation in these groups may be warranted. Efforts to improve physical health management and suicide risk reduction needs to be heightened for these groups before, during and after surges of COVID-19 infection and COVID-19 related public health interventions, such as lockdown. There is evidence that during the pandemic, a lack of parity of esteem between mental and physical health conditions may have led to staff having reduced access to personal protective equipment (PPE), COVID-19 testing and other interventions to protect and minimise the risk of COVID-19 transmission and reduce the risk of deaths in mental health or group care settings. Our findings confirm a need to ensure that people living with mental health conditions and intellectual disabilities are given equal priority to people living with physical health conditions, even during public health emergencies. These findings suggest that people living with mental disorders and intellectual disabilities may constitute a particularly vulnerable population group to COVID-19 effects, which will need to be considered in future, particularly to ensure that health inequalities are not widened further in the long-term.

Alt-text: Unlabelled box

## 1 Introduction

Major concerns have been raised about the impact of the COVID-19 pandemic on people living with mental disorders and intellectual disabilities. Prior to the pandemic, people with severe mental disorders were known to experience a 15-20 year reduction in life expectancy compared with the general population [1,2], with most causes of death attributable to preventable physical causes [3], [4], [5]. People with mental disorders have been reported to have an increased risk of COVID-19 infection [6,7], as well as hospitalisation [6] and COVID-19 mortality [6], [7], [8]. However, despite these concerns, there is little information on the impact of the pandemic on mortality risks in populations of people living with different mental disorders and intellectual disabilities. In the UK, people of Black and Asian ethnicity have higher rates of COVID-19 infection, and are more likely to experience adverse consequences, such as hospitalisation and deaths [9,10]. There are reports suggesting that ethnic inequalities in mortality across specific mental disorders have been exacerbated [11]; however, comparative evidence in this respect is scarce, which is a concern as severe mental illnesses in the UK also have a higher incidence in ethnic minority groups [12].

Following the declaration by the World Health Organization (WHO) that the novel coronavirus outbreak constituted a public health emergency of international concern on January 30^th^ 2020, the UK entered its first lockdown on March 23^rd^ 2020, with a peak in deaths in the general population noted soon after. The impact of the ‘first wave’ of COVID-19 infections and deaths on further elevating mortality risk in people with mental disorders and intellectual disabilities is unclear. Understanding mortality trends in these populations could play an important role in informing public mental health policy, for example, in determining whether particular groups may need consideration for vaccine priority and in determining impacts on other causes of death. To address this gap in evidence, we used prospective data from one of Europe's largest secondary mental healthcare providers to assess the excess risk of mortality across nine conditions (schizophrenia-spectrum disorders, affective disorders, somatoform/ neurotic disorders, personality disorders, learning disabilities, eating disorders, substance use disorders, pervasive developmental disorders, dementia). We sought to address the following research questions:

1. How has the pandemic affected excess mortality in people living with a range of different mental disorders and intellectual disabilities?
2. Has the pandemic exacerbated ethnic inequalities in mortality in people living with mental disorders and intellectual disabilities?

## 2 Methods

### 2.1 Participants and setting

The South London & Maudsley NHS Foundation Trust is one of Europe's largest secondary mental healthcare providers, providing near-complete mental healthcare to a geographically distinct and ethnically diverse catchment area in southeast London with approximately 1.36 million residents. Since 2006, health records within the Trust have been fully digital [13]. The Clinical Record Interactive Search (CRIS) system, established in 2008, is an ethically approved electronic health records interface which allows researchers to access de-identified electronic health records data from the Trust [13]. We created cohorts of people with defined and intellectual disability diagnoses followed from date of diagnosis until death or the end of the study. All people entering the cohort were alive on 1^st^ January 2019 and followed until their date of death or 31^st^ December 2020.

### 2.2 Measures

#### 2.2.1 Demographic covariates

In order to protect confidentiality, birth date of service users was provided as the first day of the birth month of the birth year and used to calculate age per quarter in this analysis. Gender was used for the adjustment by secondary standardisation. Ethnicity was classified according to Office for National Statistics criteria, comprising White British, Black Caribbean, Black African, Bangladeshi, Indian, Pakistani, and Irish groups. A ‘South Asian’ ethnicity group was created, combining the Bangladeshi, Indian and Pakistani ethnicity groups, due to small numbers. The ‘Chinese’ ethnicity group was removed due to small numbers and the ‘other’ group removed as considered heterogenous.

#### 2.2.2 Psychiatric diagnoses

Clinicians and mental health teams are required to assign diagnoses according to the *International Classification of Mental and Behavioural Disorders-10* (ICD-10) [14] to individuals who make contact with the mental health service. In the current study, we identified individuals with major psychiatric diagnoses using a combination of information from structured diagnostic fields, which were supplemented by a natural language processing (NLP) application developed with Generalised Architecture for Text Engineering (GATE) software [13], which extracts diagnostic statements from free text case note and correspondence fields. A recent audit of the performance of these NLP algorithms for clinical diagnoses found precision (positive predictive value) ranging from 0.97 to 1.00 across diagnoses at annotation and patient levels. Psychiatric diagnoses, selected according to ICD-10 chapter codes, used for the present study were: dementia (ICD-10 codes: F00-F09), mental and behavioural disorders due to substance use (ICD-10 codes: F1*), schizophrenia-spectrum disorders (ICD-10: F2*), affective disorders (including depression and bipolar disorders) (ICD-10: F3*), neurotic/ stress-related and somatoform disorders (including anxiety and adjustment disorders) (ICD-10: F4*), eating disorders (ICD-10: F50.0-F50.9), personality disorders (ICD-10: F60-F69), learning disabilities (ICD-10: F7*) and pervasive developmental disorders, such as autism (ICD-10: F8*).

### 2.3 Calendar year quarters

Cut-off points for quarters across 2019/ 2020 were at weeks 13, 26, 39, and at the end of the year (week 52). We used dates closest to the end of the quarter, on the Friday of that week. This led to the following dates being used to demarcate each quarter: 1^st^ Jan to 29 March 2019; 30 March to 28^th^ June 2019; 29 June to 27^th^ September 2019; 28^th^ September to 27^th^ December 2019; 28^th^ December 2019 to 27^th^ March 2020; 28^th^ March to 26^th^ June 2020; 27^th^ June 2020 to 25^th^ September 2020; 26^th^ September 2020 to 25^th^ December 2020. Quarter cut-off points according to these dates were applied in an identical manner to the observed and reference populations.

### 2.4 Mortality

In the UK, National Health Service (NHS) numbers are unique identifiers which link to health records and contain details, such as name, address and date of birth. The mental health Trust is notified weekly regarding deaths (with dates) of any patients who have previously or currently been in contact with these services, via the NHS ‘spine’, which allows secure information sharing across services by NHS number. Deaths notified in this way were for all-cause mortality. The dataset was extracted on 17^th^ May 2021; as there may be up to a 20-day lag in this notification process [15], this ensured that deaths had been accurately notified up to 31^st^ December 2020. For this analysis, we focussed on all-cause mortality occurring at any point from 1^st^ January 2019 to 31^st^ December 2020. Using a linkage to death certificates, deaths where COVID-19 was mentioned anywhere on the death certificate (ICD-10 codes U07.1 or U07.2) versus deaths from ‘all other causes’ were also assessed. Death registration date from linked Office for National Statistics (ONS) mortality extracts, were used in these analyses. Deaths by cause registered in each quarter (through ONS-linked data flows) differed from all-cause mortality identified through the NHS-spine due to delays in registration [16].

### 2.5 Statistical methods

For each of the diagnostic groups, we created ‘virtual’ cohorts, comprising all service users alive on 1^st^ January 2019, with start date specified as time of diagnosis, and end date specified as either death or end of the quarterly window. If service users had more than one psychiatric diagnosis at any point, they were added to cohorts for each of the diagnoses, with date of diagnosis for the first mentioned diagnosis used. Age in ten-year bands (15-24, 25-34, 35-44, 45-54, 55-64, 65-74, 75-84, 85+) was calculated by the date at midpoint of the quarter window subtracting the date of birth, and cohorts were stratified by gender (male/ female). The denominator for each quarter in the observed population was the total interval population stratified by age and gender. Observed deaths in each of the target populations (‘observed deaths’ defined as occurring in each psychiatric diagnostic group) were assessed in each quarter. If an individual died during that quarter, he or she was removed from the population for the next quarter. To estimate age- and gender-standardised mortality ratios (SMRs), we used the indirect method of standardisation, whereby we compared deaths with age and gender structure adjusted in the target (‘observed’) population to a reference population. We assessed SMRs for each mental disorder and then conducted analyses by all diagnostic groups combined (referred to as ‘any’ psychiatric diagnosis, taking the earliest recorded psychiatric diagnosis and the respective date for that diagnosis). SMRs for any psychiatric diagnosis were then assessed by ethnicity. We used five-year average weekly deaths (2015-2019) from England and Wales [17] with mid-year population estimates in 2019 [18] to estimate the ‘expected’ number of death for our target populations. We retained the same reference standard from 2019 for all analyses, to detect the changes over quarters from 1^st^ January 2019 through to December 31^st^, 2020. We then assessed age and gender-standardised mortality ratios in the general population from London, using five-year average weekly deaths (2015-2019) from England and Wales with mid-year population estimates from 2019 as the standard. This was done to illustrate the rise in relative mortality during the first wave in the general population from London, which included the catchment of the study population (the study population was in the London boroughs of Lambeth, Southwark, Croydon and Lewisham), although SMRs from the general population in London may not be comparable to SMRs for mental disorders [19]. Finally, we assessed cause-specific SMRs (deaths from COVID-19 versus all other causes) by psychiatric diagnoses and ethnicity. These were age and gender-standardised to the general population in London, using mid-year population estimates for 2019 and projected population estimates for 2020. On all graphs we have denoted a red dashed line to indicate 30^th^ January 2020, when the World Health Organization (WHO) declared COVID-19 to be a public health emergency of international concern. Stata 15.1 MP was used for all analyses, using the *istdize* suite of commands to estimate standardised mortality ratios with 95% confidence intervals.

We assessed whether deaths over quarters in the ‘pandemic period’ (2020) were greater than deaths in equivalent quarters in the ‘pre-pandemic’ period (2019) by fitting two-level random-intercepts Poisson models using the *mepoisson* command, with outcome specified as ‘observed’ deaths and offset specified as log ‘expected’ deaths, based on age and sex- adjusted rates in the reference standard [20]. Likelihood ratio tests were used to assess time*diagnosis and time*ethnicity interactions.

### 2.6 Sensitivity analyses

We conducted a series of additional sensitivity analyses to assess for sources of bias. First, we repeated all analyses which had standardised against the general population in England and Wales, by standardising against population data from London, the catchment area of the study. These analyses were conducted to assess if estimates were residually confounded by local area-level effects.

If service users had multiple diagnoses, they could appear in more than one of the cohorts. Therefore to assess the impact of this, we conducted a further sensitivity analysis, whereby we re-estimated SMRs after removing comorbid psychiatric diagnoses, assessing the impact of this in the most common condition (affective disorders).

**Fig. 1:**
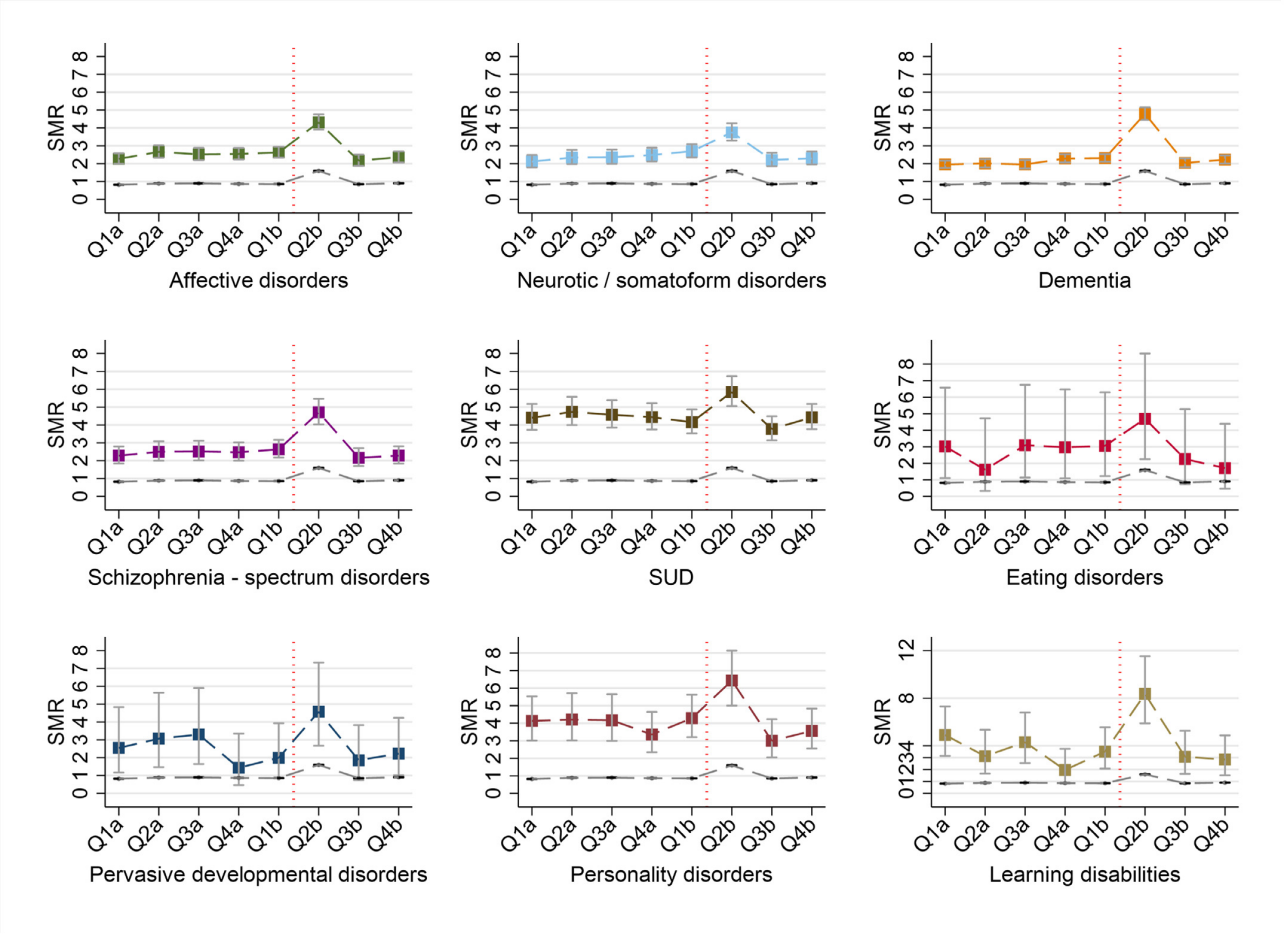
Age- and gender-standardised mortality ratios (SMRs) by psychiatric diagnoses with deaths from 2019 to end 2020?>

**Legend:** Standardised to five-year average weekly deaths (2015-2019) and mid-year population (2019) in England and Wales. a: 2019; b: 2020; Q1: quarter 1; Q2: quarter 2; Q3: quarter 3; Q4: quarter 4; SUD: Substance use disorders; Vertical red line indicates 30^th^ January 2020, when the WHO declared COVID19 a public health emergency of international concern; Coloured square markers indicate SMRs for psychiatric diagnoses; dashed grey line indicates age and gender standardised mortality ratios for general population (London); SMR: Standardised mortality ratio. Note vertical axis for learning disabilities plot ranges up to 12. See supplementary table 1 for estimates.

### 2.7 Role of the funding source

The funders did not play any role in study design, data collection, data analysis, interpretation or writing of the report.

## 3 Results

A total of 167,122 individuals with the component mental disorder diagnoses contributed data to the analyses (supplementary Figure 1; Table 1). The mean age of the combined sample was 44 years, 48.0% of the sample was male, and 45% were White British. The sample also included people of Irish (1.9%), Black Caribbean (11.6%), Black African (5.9%) and South Asian (2.6%) ethnicity. 40.0% of the sample had been diagnosed with affective disorders, 34.7% neurotic/ stress-related and somatoform disorders, 22.5% substance use disorders and 15.8% schizophrenia-spectrum disorders. All-cause mortality between 1st January 2019 and 31st December 2020 was 4.0% overall, with deaths in 1.9% (n=3227) of the sample prior to 30^th^ January 2020 (when the WHO declared a public health emergency of international concern) and deaths in 2.1% of the sample (n=3436) after 30^th^ January 2020.

**Table 1:** Demographic characteristics of the sample (N = 167,122)

	**N**	**(%)**
**Age**^1^ (years old)	43.8	18.7
**Sex**		
Female	86934	52.0%
Male	80188	48.0%
**Ethnicity**		
White British	75795	45.4%
Irish	3118	1.9%
Black Caribbean	19427	11.6%
Black African	9811	5.9%
South Asian	4405	2.6%
Other^2^/ Missing	54,566	32.6%
D**iagnoses**		
Dementia	12022	7.2%
Substance use disorders	37682	22.5%
Schizophrenia-spectrum	26368	15.8%
Affective disorders	66796	40.0%
Neurotic/ stress related & somatoform disorders	58034	34.7%
Eating disorders	9351	5.6%
Personality disorders	16442	9.8%
Learning disabilities	6045	3.6%
Pervasive developmental disorders	12489	7.5%
**Deaths**^3^	6663	4.0%

Key

1 on 1^st^ January 2019

2 Includes Chinese ethnicity, other ethnicity, other White and other mixed ethnicity groups (n=28,761; 17.2%) and missing ethnicity (n=25,805; 15.4%)

3 Deaths from 1^st^ January 2019 to 31^st^ December 2020

**Fig. 2:**
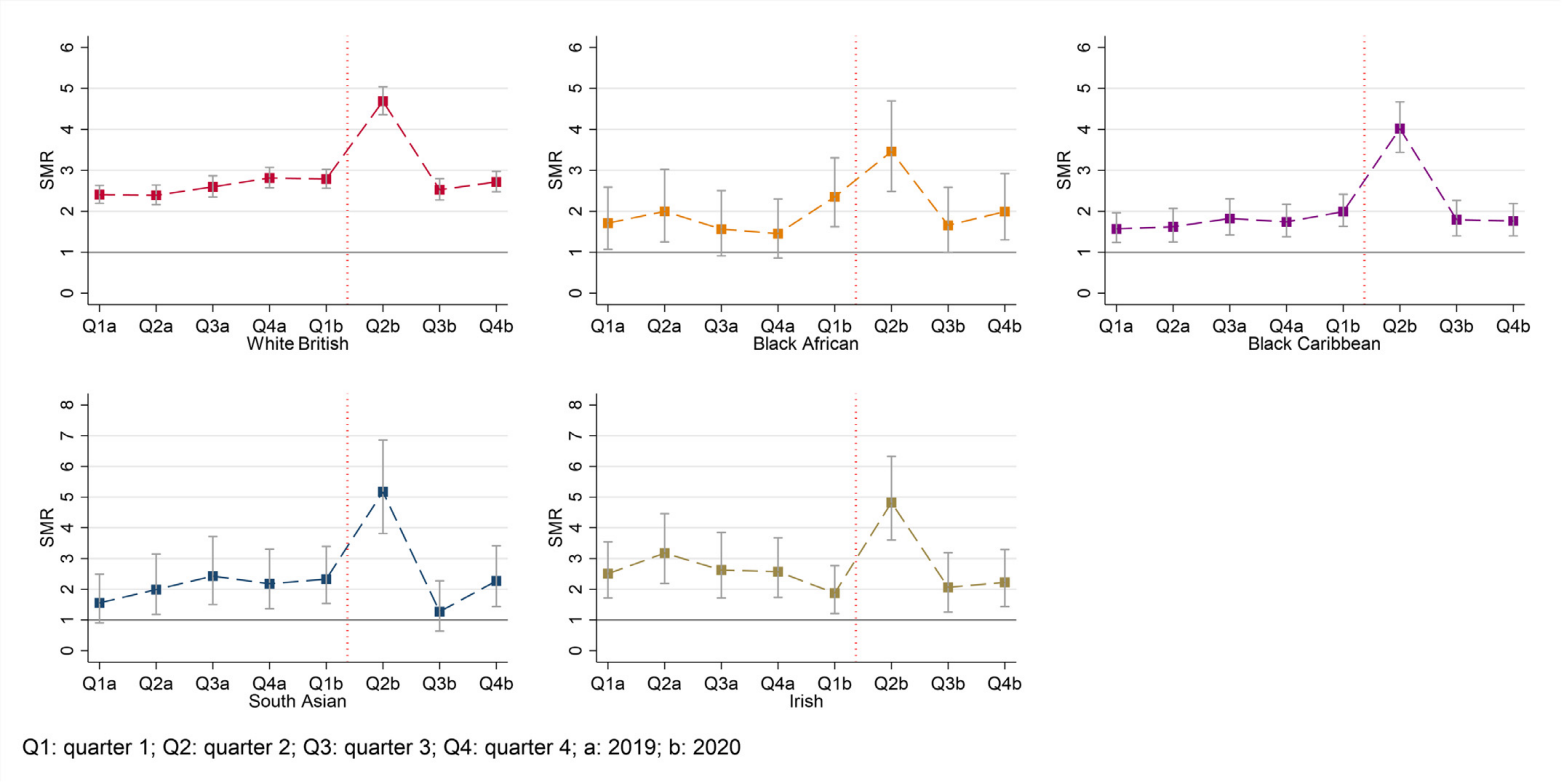
Age- and gender-standardised mortality ratios (SMRs) by ethnicity across all psychiatric diagnoses, with deaths from 2019 to end 2020?>

**Legend:** Standardised to five-year average weekly deaths (2015-2019) and mid-year population (2019) in England and Wales. Q1: quarter 1; Q2: quarter 2; Q3: quarter 3; Q4: quarter 4; a: 2019; b: 2020. Red vertical line indicates 30^th^ January 2020, when the WHO declared COVID19 a public health emergency of international concern. SMR: Standardised mortality ratio. Grey horizontal line indicates SMR of 1.00 (no difference compared to the standard/ reference population). Note vertical axes for plots for South Asian and Irish ethnicity range up to 8. See supplementary table 2 for estimates.

**Fig. 3:**
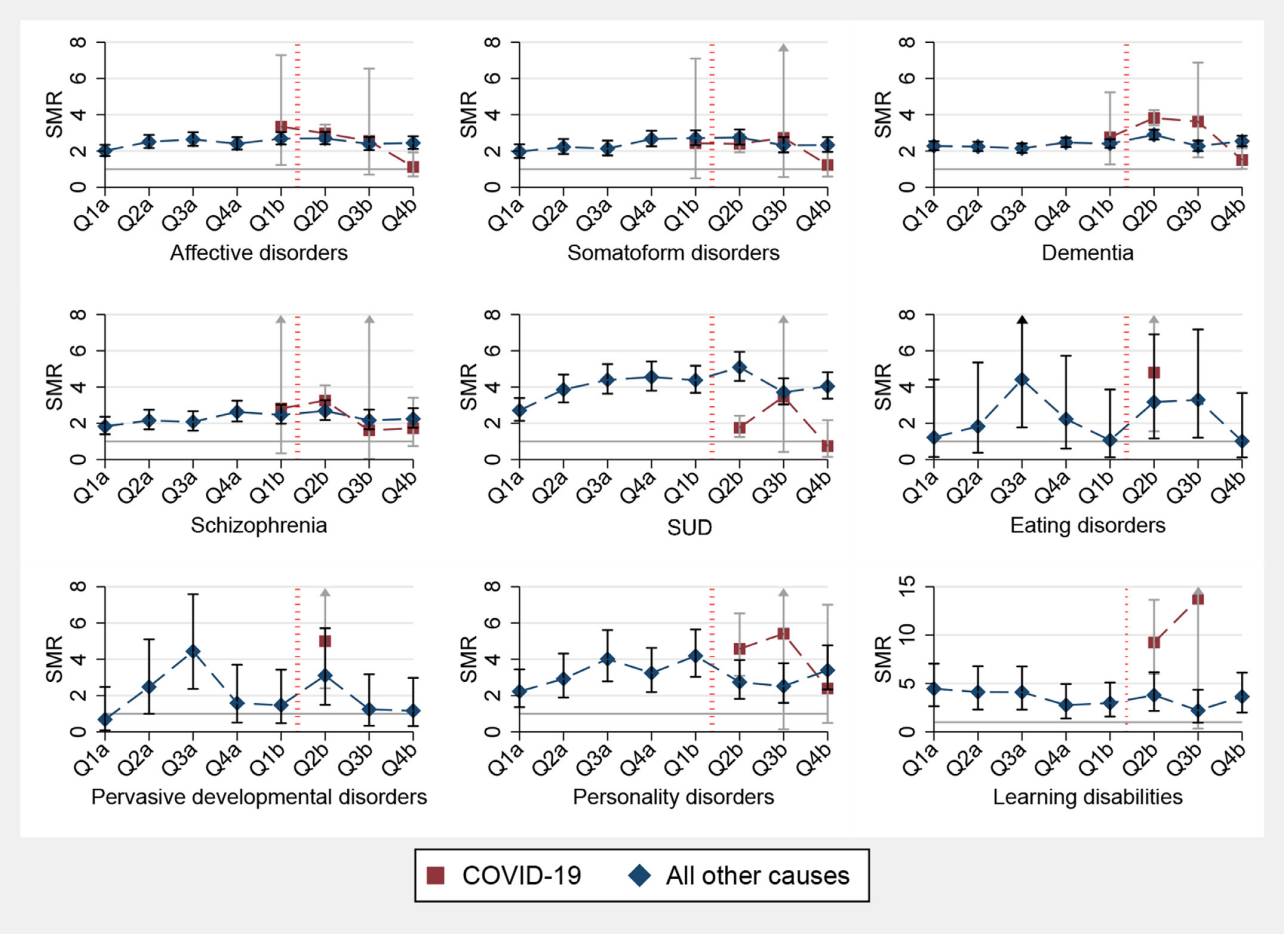
Age and gender standardised mortality ratios by cause (deaths from COVID-19 and deaths from all other/ non-COVID-19 causes over 2019-2020) by mental disorder diagnoses?>

**Fig. 4:**
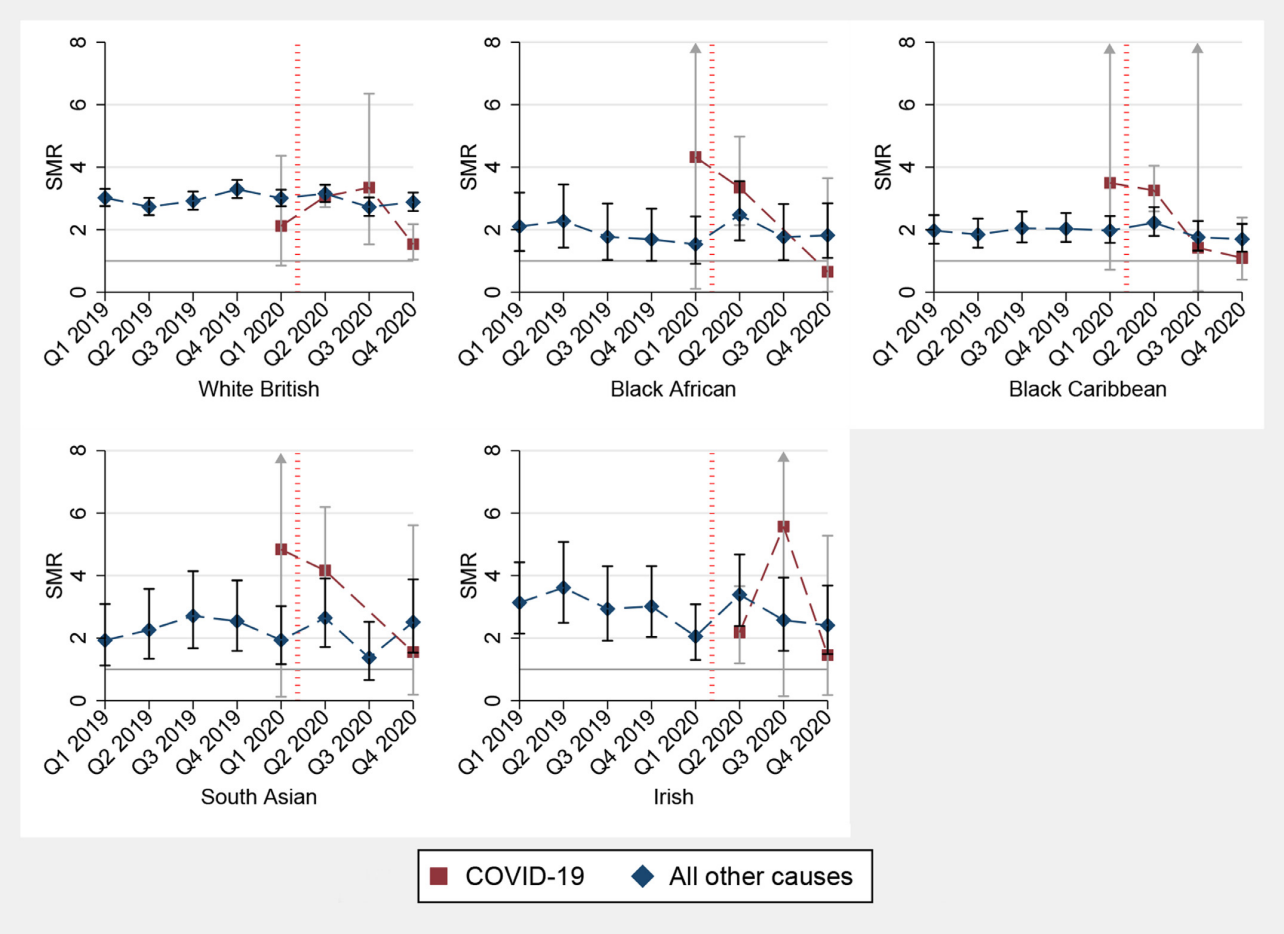
Age and gender standardised mortality ratios by cause (deaths from COVID-19 and deaths from all other/ non-COVID-19 causes over 2019-2020) by ethnicity, all mental disorders combined?>

To address the research questions, SMRs for deaths from all-causes and by cause have been plotted by diagnostic group in Figs. 1 and 3, and by ethnicity in Figs. 2 and 4. Relative to the general population in England and Wales, elevated all-cause mortality was observed across all diagnostic groups prior to the start of the pandemic (Fig. 1). Age- and gender-adjusted SMRs were then further elevated in the second quarter of 2020, across all groups (Fig. 1). A ‘peak’ in COVID-19 infection and all-cause mortality was evident in the UK general population over the same period [21], which was also evident in the general population in London (the catchment area for the study) and has been illustrated in Fig. 1 for comparison. This was an SMR of 1.60 (95% CI: 1.57-1.62) in quarter 2 of 2020 for the general population in London. Across all diagnostic groups, following observed peaks in all-cause mortality risk in quarter 2 of 2020, SMRs then returned to levels similar in magnitude to those in 2019, remaining substantially elevated relative to the general population. These trends in all-cause mortality persisted when standardised to data from London (deaths and mid-year population in London in 2019), in sensitivity analyses (see supplementary figure 2). A Likelihood ratio test to assess effect modification by mental disorder diagnosis for observed deaths over quarters in 2019/ 2020 had a p-value of 0.051. All-cause mortality in people with dementia and learning disabilities were more than double in the second quarter of 2020 compared to the equivalent quarter of 2019 (supplementary table 3). An elevated risk of all-cause mortality was also evident across substance use disorders, schizophrenia-spectrum, affective, neurotic/ somatoform and personality disorders in the second quarter of 2020 compared to the same quarter in 2019 (supplementary table 3).

Assessment of SMRs by psychiatric diagnoses disaggregated by ethnicity also revealed an increase in mortality risk in the second quarter of 2020 across all ethnic groups, including the White British group, relative to the general population (Fig. 2). Following the peak in quarter 2 of 2020, SMRs across all mental disorder groups by ethnicity reduced back to pre-existing levels, which were of a similar magnitude to those noted in 2019, and which had already been elevated relative to the general population (Fig. 2). These trends in all-cause mortality also persisted in sensitivity analyses when age and gender standardised to data from London (see supplementary figure 3). Likelihood ratio tests did not suggest effect modification by ethnicity for deaths over quarters (p=0.55), suggesting all-cause mortality trends were similar across ethnic groups. Compared to the equivalent quarter in 2019 (pre-pandemic period), all-cause mortality in quarter 2 of 2020 was elevated 2.60 times (95% CI: 1.51-4.46) in South Asian people with mental disorders and 2.48 (95% CI: 1.86-3.30), 1.96 (95% CI: 1.73-2.22), and 1.73 (95% CI: 1.03-2.91) times in Black Caribbean, White British and Black African people with mental disorders, respectively (supplementary table 3).

Fig. 3 displays SMRs for deaths from COVID-19 and from all other (non-COVID-19) causes, by diagnoses. SMRs in these figures were derived through age and gender standardisation by cause (deaths from COVID-19 and deaths from all other/ non-COVID-19 causes) to data from the general population in London, at the same time points. Across most diagnoses, the excess risk of mortality from all other causes was at least double the population average, remaining at this level throughout the pandemic period. For substance use disorders, the SMR for deaths from ‘all other causes’ rose to 5.09 (95% CI: 4.34, 5.94) in the second quarter of 2020. For some conditions (dementia, personality disorders and learning disabilities) SMRs were higher for COVID-19 related mortality compared to other causes of death. In quarter 2 of 2020 (during the initial wave of infection and deaths in the UK) COVID-19 SMRs were: 3.82 (95% CI: 3.42-4.25) for dementia, 3.26 (95% CI: 2.55-4.10) for schizophrenia-spectrum disorders, 4.81 (95% CI: 1.56-11.22) for eating disorders, 5.01 (95% CI: 2.40-9.20) for pervasive developmental disorders, 9.24 (95% CI: 5.98-13.64) for learning disabilities and 4.58 (95% CI: 3.09-6.53) for personality disorders. By the last quarter of 2020 COVID-19 SMRs were no longer elevated across most diagnoses except for dementia, where an elevated SMR for COVID-19 mortality was still evident (SMR: 1.50 (95% CI 1.02-2.15).

**Key:** Q1: quarter 1; Q2: quarter 2; Q3: quarter 3; Q4: quarter 4; a: 2019; b: 2020. SUD: Substance use disorders; Age and gender Standardised to deaths/ population in London over 2019/2020;Grey horizontal line indicates SMR of 1.00 (i.e.no difference compared to the standard/ reference population). Red vertical dashed line indicates 30^th^ January 2020, when the WHO declared COVID19 a public health emergency of international concern. SMR: Standardised mortality ratio. Note vertical axis for learning disabilities plot ranges up to 15. See supplementary table 4 for estimates.

Age and gender-standardised mortality ratios by cause, and by ethnicity, are displayed in Fig. 4. These estimates were also standardised to data from the general population in London, at the same time points. Relative to the general population, SMRs from all other causes were elevated across all ethnic groups in the pre-pandemic period (2019), continuing into the pandemic period (2020). SMRs for COVID-19 mortality were substantially elevated in quarter 2 of 2020 for the South Asian group with mental disorders (SMR: 4.16 (95% CI: 2.67-6.19) and notable for other groups (Black African: 3.35 (95% CI: 2.14-4.98), Black Caribbean: 3.26 (95% CI: 2.59-4.05), White British: 3.06 (95% CI: 2.72-3.43) and Irish: 2.18 (95% CI: 1.19-3.66). SMRs were higher for COVID-19 mortality compared to other causes of death in the Black African, Black Caribbean and South Asian groups in quarters 1 and 2 of 2020.

**Key:** Q1: quarter 1; Q2: quarter 2; Q3: quarter 3; Q4: quarter 4; Age and gender Standardised to deaths/ population in London over 2019/2020. Grey horizontal line indicates SMR of 1.00 (i.e.no difference compared to the standard/ reference population). Red vertical dashed line indicates 30^th^ January 2020, when the WHO declared COVID19 a public health emergency of international concern. SMR: Standardised mortality ratio. See supplementary table 2 for estimates.

In final sensitivity analyses, we re-estimated SMRs after removing comorbid psychiatric diagnoses for the most common condition (affective disorders). A similar trend in SMRs over time in the ‘pure’ affective disorders group compared to affective disorders with other psychiatric diagnoses was observed, but with slightly lower SMRs at each time point in the group without comorbidities. 95% CIs for estimates overlapped for the two groups (supplementary figure 4).

## 4 Discussion

Our analyses identified several findings. First, people living with a range of mental disorders, and with intellectual disabilities, had a substantially elevated risk of death in the pre-COVID-19 pandemic period, the excess risk of death continued into the pandemic period. The magnitude of elevated risks of death across mental disorders and intellectual disabilities, as observed in this study, are consistent with previously published evidence [4,5,22,23]. Second, during the first UK lockdown (declared by the UK government on 23^rd^ March 2020) and subsequent ‘wave’ of COVID-19 infection in quarter 2 of 2020, there was a sharp rise in age- and gender- standardised mortality ratios in people living with mental disorders and intellectual disabilities, when compared to data from previous years/ the pre-pandemic period. Age- and gender- standardised mortality ratios returned to pre-pandemic levels (which continued to be elevated relative to the general population) following the first ‘wave’ of COVID-19 infection and deaths in the UK.

Our analysis of cause-specific mortality sheds light on this. Deaths from all other (non-COVID-19) causes remained at least double the population average across all mental disorders and intellectual disabilities throughout the pandemic period. In contrast there was a steep rise in age and gender-standardised mortality ratios due to COVID-19, in the second and third quarters of 2020. This is noteworthy as COVID-19 mortality in the general population at this time was considerable [21], and COVID-19 SMRs across most psychiatric diagnoses were at least double or triple the population average at this time.

We also found that the increase in all-cause SMRs during the pandemic was similar across White British and ethnic minority groups in this study. In the second quarter of 2020, age and gender-standardised mortality ratios for COVID-19 increased more than two to three times the population average in Black Caribbean, Black African, White British and Irish groups with mental disorders and more than four times the population average in South Asian people with mental disorders. Deaths from all other (non-COVID-19) causes remained elevated throughout the pandemic across all ethnic groups, remaining similar compared with pre-pandemic levels. In previous work, people with mental disorders have been noted to experience shortened life expectancy lower than the general population living in deprived areas, with similar reductions in life expectancy also noted across minority ethnic groups with mental disorders [2]. The present analysis is consistent with this previous work, indicating that mental disorders have a major impact on mortality risk, with inequalities similarly experienced in White British and minority ethnic groups.

Our study provides evidence in support of the concern that the COVID-19 pandemic has exacerbated pre-existing health inequalities in people living with mental disorders and intellectual disabilities, suggesting that additional excess mortality has been driven by a heightened risk of death from COVID-19, added to elevated risks of death from all other causes, which have continued to operate throughout the pandemic.

Strengths of our study included our use of prospective real-time health records with ‘live’ notification of deaths in the records. As deaths were notified via the NHS spine point, we were able to assess this in all people who had ever been in contact with services, even if they had been discharged or moved, ensuring ascertainment of mortality was at high levels of completeness. The use of health records also meant that we were able to extract and analyse data rapidly. As the pandemic progresses, we will be seeking to re-run analyses to provide further rapid intelligence on the nature of mortality in mental disorders. Our findings, which indicate the dynamic nature of these trends, particularly for COVID-19 mortality, highlight a need for similar systems to be potentially accessible across the UK and in other countries. There have been major concerns that people of an ethnic minority background are at a higher risk of COVID-19 infection and mortality [9,10], and ethnic minority groups within the mental health system are more likely to experience inequalities in accessing evidence-based treatments [24]. It therefore remains imperative that ethnic inequalities relating to mortality are assessed wherever possible. Our study therefore has a further strength that, given the coverage of the study over an ethnically diverse catchment area in London, UK, where data collection on ethnicity is largely a mandatory and routinely collected data field within the health records, assessment by ethnicity was feasible. Finally, we standardised mortality estimates in our sample to data from London, the catchment of the study. This permitted an assessment against regional trends, which differed from national trends during the first wave [21].

There are several limitations. First, although we were able to assess cause of death through linked death certificate information, it was not possible to assess mortality due to ‘natural’ or ‘unnatural’ causes, as the equivalent comparison data from the general population, was not available at the time of analysis [25]. Our analyses on cause-specific mortality used cause of deaths registration data which may be subject to delays, although deaths from COVID-19 have been noted to be registered more quickly than deaths from all other causes [16].

Although we were able to assess mortality risk by ethnicity, psychiatric diagnoses were based on clinician judgement. Concerns have previously been raised that racially biased practices may lead to Black people being more likely to be diagnosed with psychotic or more ‘severe’ diagnoses that would not otherwise meet research criteria. If this were the case, we might anticipate that our analyses would have underestimated mortality risk for these groups (and therefore SMRs would be even higher than the estimates which we have provided in this report) since such biased practices would have meant that people with less severe mental health problems would have been included in these groups. Our analyses of ethnicity were also limited by smaller numbers in some specific groups and may have hampered assessment of interactions, due to lower power. This also limited us from being able to further address the issue of intersectionality by ethnicity and mental health diagnosis, or indeed by other aspects such as gender [26]. Future work may aim to address this. As the mental health Trust provides near-complete secondary mental healthcare to all people resident in the catchment area (c. 1.36 million people), we can be reasonably certain that people requiring secondary mental healthcare (care provided by specialist mental health services rather than primary care only) would have been represented in the study population. However, our assessment of mortality risk was therefore representative of people in contact with secondary mental healthcare services, and therefore living with mental disorders and intellectual disabilities, which were more debilitating and severe. The findings in this study may not be generalisable to people living with less severe conditions not requiring secondary mental healthcare. It is a limitation that we could not account for indicators such as area-level deprivation or individual-level socioeconomic position in the present analyses, particularly as parts of the catchment area for the study also fell within some of the most deprived areas in England, therefore estimates may have been residually confounded by socioeconomic position. However, by standardising to data from the local catchment area from London, residual confounding effects from area-level deprivation would have been reduced. Although the catchment area of the present study may reflect other similar urban or metropolitan regions across the UK, generalisability of the present study may also be restricted to UK urban areas and future work should aim to explore regional variability as well as urban-rural differences.

## 5 Relationship to previous work and implications

Previous studies have suggested that the risk of COVID-19 infection is elevated in people with mental disorders [6], and studies from the US, Denmark, South Korea and the UK have also confirmed elevated hospitalisation and mortality risk in the initial few weeks following COVID-19 infection, in people living with a range of psychiatric diagnoses [[6], [7], [8],[27], [28], [29]]. Some evidence of ethnic inequalities in mortality risk in the UK, has been described in people living with learning disabilities [11]. In many countries, the pandemic has led to disruptions in routine healthcare, potentially leading to delayed presentation to services for acute physical conditions. Our study suggests a dynamic nature for observed trends, accounted for by deaths from COVID-19, on a background of ongoing excess mortality from other (non-COVID-19) causes. Excess risk of deaths from COVID-19 and from other/ non-COVID-19 related causes may have been through a number of factors, including a higher prevalence of underlying long-term physical health comorbidities known to put people at an excess risk of preventable mortality [1], [2], [3], [4], [5] which may also increase the risk of serious complications or death following COVID-19 infection [10,28]. Potential inequalities and delays impacting accessing care for COVID-19 infection may have also contributed [30]. Disruption in access to routine healthcare as a result of lockdown may have also played a role [21]. Concerns have also been raised that people residing in group situations [11] or admitted to inpatient psychiatric units [30] may have had an increased risk of contracting COVID-19 infection, exacerbated by shortages in personal protective equipment (PPE) or delayed access to testing [30]. Finally, public health interventions, such as lockdown and social distancing, may have exacerbated social isolation and disrupted social networks, which may add risks for mental health relapse, associated with adverse outcomes such as suicide. However, to date increases in suicidality have not been noted as a result of the pandemic, with stability in prevalence of suicidal ideation and deaths noted in the first six months of the pandemic, compared to pre-pandemic prevalence [31]. We were unable to assess suicide mortality in the current analysis however this underlying causes of excess mortality will require further urgent investigation.

The findings indicate that people with mental disorders and intellectual disabilities experienced substantial increases in mortality risks which were already significantly elevated compared with the general population prior to the outbreak of the pandemic, with dynamic additional increases, due to the impact of COVID-19 infection and death. As the pandemic progresses, our findings suggest that prioritised vaccine access may be needed for these groups internationally. In addition to prioritisation, implementation and supporting decision-making in people with mental disorders and intellectual disabilities, who may have many additional risk factors for vaccine hesitancy will need to be considered [32], and research relating to good practice in promoting high levels of vaccine uptake is needed [33]. Approaches to optimise physical health care and suicide risk reduction, before, during and after peaks of COVID-19 infection to prevent further excess mortality in people living with mental disorders and intellectual disabilities will also be needed.

## Contributors

JD conceived the study and led all analyses. All authors contributed to the design of the work, MB supported acquisition of data and all authors contributed to analysis and interpretation. All authors contributed to the drafting of the work and revising it critically for intellectual content and have approved the final version to be published. All authors agree to be accountable for all aspects of the work in ensuring that questions related to the accuracy or integrity of any part of the work are appropriately investigated and resolved. JD is guarantor of all analyses.

## Declaration of competing interest

RS has received research support in the last 3 years from Janssen, GSK and Takeda.

## Appendix Supplementary materials

Image, application 1

Image, application 2

## Footnotes

Supplementary material associated with this article can be found in the online version at https://doi.org/10.1016/j.lanepe.2021.100228.

## References

[1] Hjorthøj C. (2017). Years of potential life lost and life expectancy in schizophrenia: a systematic review and meta-analysis. Lancet Psychiatry.

[2] Das-Munshi J. (2020). How do ethnicity and deprivation impact on life expectancy at birth in people with serious mental illness? Observational study in the UK. Psychol Med.

[3] Lawrence D., Hancock K.J., Kisely S. (2013). The gap in life expectancy from preventable physical illness in psychiatric patients in Western Australia: retrospective analysis of population based registers. BMJ.

[4] Das-Munshi J. (2017). Ethnicity and excess mortality in severe mental illness: a cohort study. Lancet Psychiatry.

[5] Das-Munshi J. (2019). Depression and cause-specific mortality in an ethnically diverse cohort from the UK: 8-year prospective study. Psychol Med.

[6] Yang H. (2020). Pre-pandemic psychiatric disorders and risk of COVID-19: a UK Biobank cohort analysis. Lancet Healthy Longevity.

[7] Wang Q., Xu R., Volkow N.D. (2021). Increased risk of COVID-19 infection and mortality in people with mental disorders: analysis from electronic health records in the United States. World Psychiatry.

[8] Li L. (2020). Association of a prior psychiatric diagnosis with mortality among hospitalized patients with coronavirus disease 2019 (COVID-19) infection. JAMA Network Open.

[9] Sze S. (2020). Ethnicity and clinical outcomes in COVID-19: a systematic review and meta-analysis. EClinicalMedicine.

[10] Public Health England (2020). https://assets.publishing.service.gov.uk/government/uploads/system/uploads/attachment_data/file/908434/Disparities_in_the_risk_and_outcomes_of_COVID_August_2020_update.pdf.

[11] Public Health England (2020). https://www.gov.uk/government/publications/covid-19-deaths-of-people-with-learning-disabilities/covid-19-deaths-of-people-identified-as-having-learning-disabilities-summary.

[12] Oduola S. (2021). Change in incidence rates for psychosis in different ethnic groups in south London: findings from the Clinical Record Interactive Search-First Episode Psychosis (CRIS-FEP) study. Psychol Med.

[13] Perera G. (2016). Cohort profile of the South London and Maudsley NHS Foundation Trust Biomedical Research Centre (SLaM BRC) Case Register: current status and recent enhancement of an Electronic Mental Health Record-derived data resource. BMJ Open.

[14] World Health Organization (2011).

[15] NHS digital. Mortality data review. https://digital.nhs.uk/coronavirus/coronavirus-data-services-updates/mortality-data-review. Accessed February 2021.

[16] Office for National Statistics, Deaths involving COVID-19, England and Wales: deaths occurring in June 2020, Fromhttps://www.ons.gov.uk/peoplepopulationandcommunity/birthsdeathsandmarriages/deaths/bulletins/deathsinvolvingcovid19englandandwales/deathsoccurringinjune2020#time-taken-for-the-deaths-in-march-to-june-to-be-registered accessed 1st June 2021. 2020.

[17] Office of National Statistics. Five year average weekly deaths by place of death, England and Wales, deaths occurring between 2015 and 2019.https://www.ons.gov.uk/peoplepopulationandcommunity/birthsdeathsandmarriages/deaths/adhocs/11622fiveyearaverageweeklydeathsbyplaceofdeathenglandandwalesdeathsoccurringbetween2015and2019. 2020 23 April 2020.

[18] Office for National Statistics. Estimates of the population for the UK, England and Wales, Scotland and Northern Ireland.https://www.ons.gov.uk/peoplepopulationandcommunity/populationandmigration/populationestimates/datasets/populationestimatesforukenglandandwalesscotlandandnorthernireland. 2020 [cited 2021.

[19] Goldman D.A., Brender J.D. (2000). Are standardized mortality ratios valid for public health data analysis?. Stat Med.

[20] STATA. Multilevel mixed effects Poisson regression; Higher level models. pg 11. https://www.stata.com/manuals/memepoisson.pdf accessed May 2021. [cited 2020.

[21] Kontopantelis E. (2021). Excess mortality in England and Wales during the first wave of the COVID-19 pandemic. J Epidemiol Community Health.

[22] Chang C.-K. (2010). All-cause mortality among people with serious mental illness (SMI), substance use disorders, and depressive disorders in southeast London: a cohort study. BMC psychiatry.

[23] Harris C., Barraclough B. (1998). Excess mortality of mental disorder. Br J Psychiatry.

[24] Das-Munshi J., Bhugra D., Crawford M.J. (2018). Ethnic minority inequalities in access to treatments for schizophrenia and schizoaffective disorders: findings from a nationally representative cross-sectional study. BMC Med.

[25] Office for National Statistics, Deaths caused by suicide by quarter in England. https://www.ons.gov.uk/peoplepopulationandcommunity/birthsdeathsandmarriages/deaths/datasets/deathscausedbysuicidebyquarterinengland Accessed 1st June 2021. 2021.

[26] Crenshaw K. (1991). Mapping the margins: intersectionality, identity politics, and violence against women of color. Stanford Law Rev.

[27] Nemani K. (2021). Association of psychiatric disorders with mortality among patients with COVID-19. JAMA Psychiatry.

[28] Reilev M. (2020). Characteristics and predictors of hospitalization and death in the first 11 122 cases with a positive RT-PCR test for SARS-CoV-2 in Denmark: a nationwide cohort. Int J Epidemiol.

[29] Lee D.Y. (2020). Risk of mortality in elderly coronavirus disease 2019 patients with mental health disorders: a nationwide retrospective study in South Korea. Am J Geriatr Psychiatry.

[30] Livingston G. (2020). Prevalence, management, and outcomes of SARS-CoV-2 infections in older people and those with dementia in mental health wards in London, UK: a retrospective observational study. Lancet Psychiatry.

[31] Knudsen A.K.S. (2021). Prevalence of mental disorders, suicidal ideation and suicides in the general population before and during the COVID-19 pandemic in Norway: A population-based repeated cross-sectional analysis. Lancet Regional Health - Eur.

[32] Warren N., Kisely S., Siskind D. (2021). Maximizing the uptake of a COVID-19 vaccine in people with severe mental illness: a public health priority. JAMA Psychiatry.

[33] Mazereel V. (2021 May). COVID-19 vaccination for people with severe mental illness: why, what, and how?. The Lancet Psychiatry.

