# Supplementary material for: All-cause and cause-specific mortality in people with mental disorders and intellectual disabilities, before and during the COVID-19 pandemic: cohort study
Source: Lancet Reg Health Eur. 2021 Oct 7;11:100228. doi: 10.1016/j.lanepe.2021.100228 (PMC8639185; doi:10.1016/j.lanepe.2021.100228)
Supplement: Supplementary file 1 [file mmc1.docx]

**SUPPLEMENTARY MATERIAL**

**TITLE:** All-cause and cause-specific mortality in people with mental disorders before and during the COVID-19 pandemic: cohort study

**AUTHORS:** Das-Munshi Jayati, Chang Chin Kuo, Bakolis Ioannis, Broadbent Matthew, Dregan Alex, Hotopf Matthew, Morgan Craig, Stewart Robert

**SUPPLEMENTARY TABLES:** 4

**SUPPLEMENTARY FIGURES:** 4

**Supplementary Figure 1:** Flow chart of study population*

**
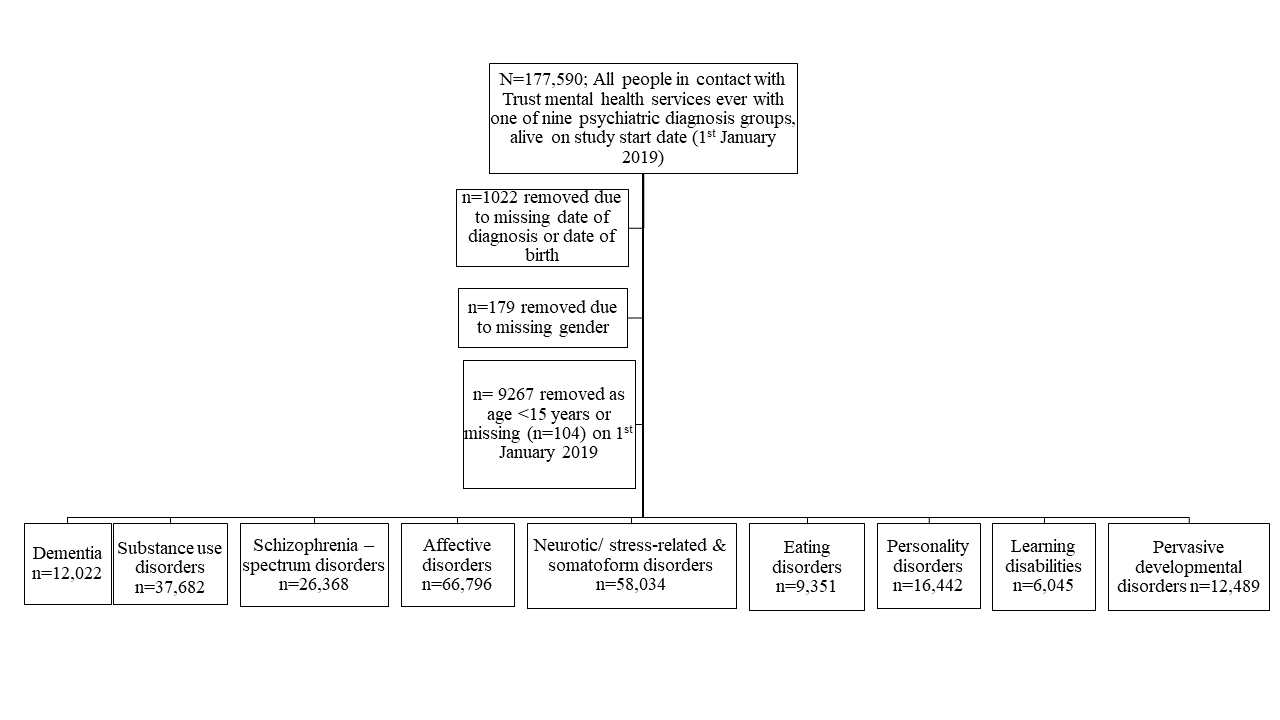
**

**Totals exclude people who opted out nationally, applied to data extractions since April 2021 as a legal requirement. This was 2.89% of the population registered with a General Practice on 1^st^ June 2021 (see ‘*[*National data opt-out, June 2021*](https://digital.nhs.uk/data-and-information/publications/statistical/national-data-opt-out/june-2021)*’).*

**Supplementary Table 1:** Age- and gender-standardised mortality ratios (SMRs) across

psychiatric diagnoses, by quarters in 2019 and 2020. SMRs for the general population

in London, over the same period is provided at the end of the table.

| **Diagnosis** | **Quarter, year** | **Observed**  **deaths** | **Expected**  **deaths** | **SMR** | **(95% CI)** | |
| --- | --- | --- | --- | --- | --- | --- |
| Substance use disorders (F1*) | Q1, 2019 | 149 | 34 | 4.41 | 3.73 | 5.18 |
|  | Q2, 2019 | 144 | 30 | 4.74 | 3.99 | 5.58 |
|  | Q3, 2019 | 142 | 31 | 4.58 | 3.85 | 5.39 |
|  | Q4, 2019 | 146 | 33 | 4.44 | 3.75 | 5.22 |
|  | Q1, 2020 | 154 | 37 | 4.16 | 3.53 | 4.87 |
|  | Q2, 2020 | 194 | 33 | 5.85 | 5.06 | 6.74 |
|  | Q3, 2020 | 127 | 34 | 3.77 | 3.15 | 4.49 |
|  | Q4, 2020 | 158 | 36 | 4.43 | 3.77 | 5.18 |
| Dementia (F0*) | Q1, 2019 | 374 | 191 | 1.96 | 1.76 | 2.17 |
|  | Q2, 2019 | 305 | 151 | 2.02 | 1.80 | 2.25 |
|  | Q3, 2019 | 275 | 140 | 1.96 | 1.73 | 2.21 |
|  | Q4, 2019 | 396 | 173 | 2.29 | 2.07 | 2.53 |
|  | Q1, 2020 | 462 | 199 | 2.32 | 2.11 | 2.54 |
|  | Q2, 2020 | 730 | 152 | 4.79 | 4.45 | 5.15 |
|  | Q3, 2020 | 273 | 134 | 2.04 | 1.81 | 2.30 |
|  | Q4, 2020 | 364 | 163 | 2.23 | 2.01 | 2.47 |
| Schizophrenia-spectrum disorders (F2*) | Q1, 2019 | 92 | 40 | 2.28 | 1.84 | 2.80 |
|  | Q2, 2019 | 86 | 34 | 2.50 | 2.00 | 3.09 |
|  | Q3, 2019 | 85 | 34 | 2.52 | 2.01 | 3.12 |
|  | Q4, 2019 | 94 | 38 | 2.47 | 2.00 | 3.02 |
|  | Q1, 2020 | 114 | 43 | 2.63 | 2.17 | 3.17 |
|  | Q2, 2020 | 172 | 37 | 4.71 | 4.03 | 5.47 |
|  | Q3, 2020 | 76 | 35 | 2.16 | 1.70 | 2.71 |
|  | Q4, 2020 | 90 | 39 | 2.29 | 1.84 | 2.81 |
| Affective disorders (F3*) | Q1, 2019 | 234 | 103 | 2.27 | 1.99 | 2.58 |
|  | Q2, 2019 | 232 | 87 | 2.66 | 2.33 | 3.03 |
|  | Q3, 2019 | 214 | 85 | 2.53 | 2.20 | 2.89 |
|  | Q4, 2019 | 246 | 97 | 2.55 | 2.24 | 2.89 |
|  | Q1, 2020 | 290 | 110 | 2.63 | 2.34 | 2.95 |
|  | Q2, 2020 | 395 | 92 | 4.32 | 3.90 | 4.76 |
|  | Q3, 2020 | 189 | 87 | 2.18 | 1.88 | 2.51 |
|  | Q4, 2020 | 233 | 99 | 2.36 | 2.07 | 2.69 |
| Neurotic, stress related and somatoform disorders (F4*) | Q1, 2019 | 149 | 70 | 2.12 | 1.79 | 2.49 |
|  | Q2, 2019 | 141 | 60 | 2.35 | 1.98 | 2.77 |
|  | Q3, 2019 | 139 | 59 | 2.37 | 1.99 | 2.79 |
|  | Q4, 2019 | 168 | 68 | 2.49 | 2.13 | 2.89 |
|  | Q1, 2020 | 210 | 78 | 2.70 | 2.35 | 3.09 |
|  | Q2, 2020 | 243 | 65 | 3.76 | 3.30 | 4.26 |
|  | Q3, 2020 | 138 | 62 | 2.21 | 1.86 | 2.61 |
|  | Q4, 2020 | 163 | 71 | 2.30 | 1.96 | 2.69 |
| Eating disorders (F50) | Q1, 2019 | <10 | ND | 3.02 | 1.11 | 6.58 |
|  | Q2, 2019 | <10 | ND | 1.62 | 0.33 | 4.72 |
|  | Q3, 2019 | <10 | ND | 3.10 | 1.14 | 6.75 |
|  | Q4, 2019 | <10 | ND | 2.97 | 1.09 | 6.47 |
|  | Q1, 2020 | <10 | ND | 3.06 | 1.23 | 6.30 |
|  | Q2, 2020 | <10 | ND | 4.70 | 2.25 | 8.64 |
|  | Q3, 2020 | <10 | ND | 2.26 | 0.73 | 5.28 |
|  | Q4, 2020 | <10 | ND | 1.71 | 0.47 | 4.39 |
| Pervasive developmental disorders (F8*) | Q1, 2019 | <10 | ND | 2.55 | 1.16 | 4.83 |
|  | Q2, 2019 | <10 | ND | 3.07 | 1.47 | 5.64 |
|  | Q3, 2019 | 11 | 3 | 3.30 | 1.65 | 5.90 |
|  | Q4, 2019 | <10 | ND | 1.43 | 0.47 | 3.35 |
|  | Q1, 2020 | <10 | ND | 1.99 | 0.86 | 3.93 |
|  | Q2, 2020 | 17 | 4 | 4.58 | 2.67 | 7.33 |
|  | Q3, 2020 | <10 | ND | 1.86 | 0.75 | 3.83 |
|  | Q4, 2020 | <10 | ND | 2.23 | 1.02 | 4.23 |
| Learning disabilities (F7*) | Q1, 2019 | 24 | 5 | 4.91 | 3.15 | 7.31 |
|  | Q2, 2019 | 13 | 4 | 3.13 | 1.67 | 5.35 |
|  | Q3, 2019 | 18 | 4 | 4.30 | 2.55 | 6.80 |
|  | Q4, 2019 | <10 | ND | 1.97 | 0.90 | 3.74 |
|  | Q1, 2020 | 18 | 5 | 3.52 | 2.08 | 5.56 |
|  | Q2, 2020 | 37 | 4 | 8.36 | 5.89 | 11.53 |
|  | Q3, 2020 | 13 | 4 | 3.08 | 1.64 | 5.26 |
|  | Q4, 2020 | 13 | 5 | 2.85 | 1.52 | 4.88 |
| Personality disorders (F6*) | Q1, 2019 | 45 | 11 | 4.13 | 3.01 | 5.53 |
|  | Q2, 2019 | 41 | 10 | 4.21 | 3.02 | 5.71 |
|  | Q3, 2019 | 41 | 10 | 4.17 | 2.99 | 5.65 |
|  | Q4, 2019 | 36 | 11 | 3.35 | 2.35 | 4.64 |
|  | Q1, 2020 | 52 | 12 | 4.29 | 3.20 | 5.63 |
|  | Q2, 2020 | 69 | 11 | 6.43 | 5.00 | 8.14 |
|  | Q3, 2020 | 32 | 11 | 3.00 | 2.05 | 4.23 |
|  | Q4, 2020 | 41 | 12 | 3.56 | 2.56 | 4.83 |
| General population | Q1, 2019 | 12986 | 15796 | 0.82 | 0.81 | 0.84 |
| (London) | Q2, 2019 | 11580 | 13043 | 0.89 | 0.87 | 0.90 |
|  | Q3, 2019 | 11159 | 12410 | 0.90 | 0.88 | 0.92 |
|  | Q4, 2019 | 12137 | 14002 | 0.87 | 0.85 | 0.88 |
|  | Q1, 2020 | 13783 | 16079 | 0.86 | 0.84 | 0.87 |
|  | Q2, 2020 | 21181 | 13278 | 1.60 | 1.57 | 1.62 |
|  | Q3, 2020 | 10763 | 12635 | 0.85 | 0.84 | 0.87 |
|  | Q4, 2020 | 12929 | 14255 | 0.91 | 0.89 | 0.92 |

***Key****: ND: Not displayed; Standardised to five-year average weekly deaths*

*(2015-2019) and mid-year population (2019) in England and Wales.*

*.*

**Supplementary Table 2:** Age- and gender-standardised mortality ratios (SMRs) by ethnicity (all diagnoses), all-cause mortality and for COVID-19 mortality and deaths from all other/ non-COVID-19 causes; Quarters in 2019 and 2020

| **Ethnicity** | **Quarter, year** | **Observed deaths** | **Expected deaths** | **SMR** | **(95% CI)** | | **Cause of death*** | |
| --- | --- | --- | --- | --- | --- | --- | --- | --- |
| White British | Q1, 2019 | 477 | 198 | 2.41 | 2.19 | 2.63 | All-cause mortality | |
|  | Q2, 2019 | 386 | 161 | 2.39 | 2.16 | 2.64 | All-cause mortality | |
|  | Q3, 2019 | 397 | 153 | 2.60 | 2.35 | 2.86 | All-cause mortality | |
|  | Q4, 2019 | 502 | 178 | 2.81 | 2.57 | 3.07 | All-cause mortality | |
|  | Q1, 2020 | 565 | 203 | 2.79 | 2.56 | 3.03 | All-cause mortality | |
|  | Q2, 2020 | 756 | 161 | 4.69 | 4.36 | 5.03 | All-cause mortality | |
|  | Q3, 2020 | 372 | 147 | 2.52 | 2.27 | 2.79 | All-cause mortality | |
|  | Q4, 2020 | 462 | 170 | 2.72 | 2.47 | 2.98 | All-cause mortality | |
|  | Q1, 2019 | 477 | 158 | 3.02 | 2.76 | 3.30 | Non COVID19 mortality | |
|  | Q2, 2019 | 386 | 141 | 2.73 | 2.47 | 3.02 | Non COVID19 mortality | |
|  | Q3, 2019 | 397 | 136 | 2.92 | 2.64 | 3.22 | Non COVID19 mortality | |
|  | Q4, 2019 | 502 | 152 | 3.29 | 3.01 | 3.59 | Non COVID19 mortality | |
|  | Q1, 2020 | 510 | 170 | 3.01 | 2.75 | 3.28 | Non COVID19 mortality | |
|  | Q2, 2020 | 513 | 163 | 3.15 | 2.89 | 3.44 | Non COVID19 mortality | |
|  | Q3, 2020 | 336 | 123 | 2.72 | 2.44 | 3.03 | Non COVID19 mortality | |
|  | Q4, 2020 | 381 | 132 | 2.88 | 2.60 | 3.19 | Non COVID19 mortality | |
|  | Q1, 2020 | <10 | ND | 2.12 | 0.85 | 4.37 | COVID-19 |  |
|  | Q2, 2020 | 293 | 96 | 3.06 | 2.72 | 3.43 | COVID-19 |  |
|  | Q3, 2020 | <10 | ND | 3.35 | 1.53 | 6.35 | COVID-19 |  |
|  | Q4, 2020 | 31 | 20 | 1.53 | 1.04 | 2.18 | COVID-19 |  |
| Black African | Q1, 2019 | 22 | 13 | 1.71 | 1.07 | 2.59 | All-cause mortality |  |
|  | Q2, 2019 | 22 | 11 | 2.00 | 1.25 | 3.03 | All-cause mortality |  |
|  | Q3, 2019 | 17 | 11 | 1.56 | 0.91 | 2.51 | All-cause mortality |  |
|  | Q4, 2019 | 18 | 12 | 1.45 | 0.86 | 2.30 | All-cause mortality |  |
|  | Q1, 2020 | 33 | 14 | 2.35 | 1.62 | 3.30 | All-cause mortality |  |
|  | Q2, 2020 | 41 | 12 | 3.46 | 2.48 | 4.69 | All-cause mortality |  |
|  | Q3, 2020 | 19 | 11 | 1.66 | 1.00 | 2.59 | All-cause mortality |  |
|  | Q4, 2020 | 26 | 13 | 1.99 | 1.30 | 2.92 | All-cause mortality |  |
|  | Q1, 2019 | 22 | 10 | 2.10 | 1.32 | 3.19 | Non COVID19 mortality | |
|  | Q2, 2019 | 22 | 10 | 2.28 | 1.43 | 3.45 | Non COVID19 mortality | |
|  | Q3, 2019 | 17 | 10 | 1.77 | 1.03 | 2.84 | Non COVID19 mortality | |
|  | Q4, 2019 | 18 | 11 | 1.69 | 1.00 | 2.67 | Non COVID19 mortality | |
|  | Q1, 2020 | 18 | 12 | 1.53 | 0.91 | 2.42 | Non COVID19 mortality | |
|  | Q2, 2020 | 29 | 12 | 2.47 | 1.66 | 3.55 | Non COVID19 mortality | |
|  | Q3, 2020 | 17 | 10 | 1.76 | 1.03 | 2.82 | Non COVID19 mortality | |
|  | Q4, 2020 | 19 | 10 | 1.82 | 1.10 | 2.84 | Non COVID19 mortality | |
|  | Q1, 2020 | <10 | ND | 4.32 | 0.11 | 8.00 | COVID-19 |  |
|  | Q2, 2020 | 24 | 7 | 3.35 | 2.14 | 4.98 | COVID-19 |  |
|  | Q3, 2020 | 0 | - | - | - | - | COVID-19 |  |
|  | Q4, 2020 | <10 | ND | 0.65 | 0.02 | 3.65 | COVID-19 |  |
| Black Caribbean | Q1, 2019 | 76 | 48 | 1.57 | 1.24 | 1.96 | All-cause mortality |  |
|  | Q2, 2019 | 65 | 40 | 1.62 | 1.25 | 2.07 | All-cause mortality |  |
|  | Q3, 2019 | 70 | 38 | 1.82 | 1.42 | 2.30 | All-cause mortality |  |
|  | Q4, 2019 | 79 | 45 | 1.74 | 1.38 | 2.17 | All-cause mortality |  |
|  | Q1, 2020 | 104 | 52 | 1.99 | 1.63 | 2.41 | All-cause mortality |  |
|  | Q2, 2020 | 170 | 42 | 4.02 | 3.44 | 4.67 | All-cause mortality |  |
|  | Q3, 2020 | 70 | 39 | 1.79 | 1.40 | 2.27 | All-cause mortality |  |
|  | Q4, 2020 | 81 | 46 | 1.76 | 1.40 | 2.19 | All-cause mortality | |
|  | Q1, 2019 | 76 | 39 | 1.97 | 1.55 | 2.47 | Non COVID19 mortality | |
|  | Q2, 2019 | 65 | 35 | 1.85 | 1.43 | 2.35 | Non COVID19 mortality | |
|  | Q3, 2019 | 70 | 34 | 2.04 | 1.59 | 2.58 | Non COVID19 mortality | |
|  | Q4, 2019 | 79 | 39 | 2.03 | 1.61 | 2.53 | Non COVID19 mortality | |
|  | Q1, 2020 | 86 | 44 | 1.97 | 1.58 | 2.44 | Non COVID19 mortality | |
|  | Q2, 2020 | 95 | 43 | 2.22 | 1.80 | 2.72 | Non COVID19 mortality | |
|  | Q3, 2020 | 57 | 32 | 1.76 | 1.33 | 2.28 | Non COVID19 mortality | |
|  | Q4, 2020 | 60 | 35 | 1.70 | 1.30 | 2.19 | Non COVID19 mortality | |
|  | Q1, 2020 | <10 | ND | 3.50 | 0.72 | 8.00 | COVID-19 |  |
|  | Q2, 2020 | 81 | 25 | 3.26 | 2.59 | 4.05 | COVID-19 |  |
|  | Q3, 2020 | <10 | ND | 1.42 | 0.04 | 7.93 | COVID-19 |  |
|  | Q4, 2020 | <10 | ND | 1.10 | 0.40 | 2.39 | COVID-19 |  |
| Irish | Q1, 2019 | 32 | 13 | 2.51 | 1.72 | 3.54 | All-cause mortality |  |
|  | Q2, 2019 | 33 | 10 | 3.18 | 2.19 | 4.46 | All-cause mortality |  |
|  | Q3, 2019 | 26 | 10 | 2.63 | 1.72 | 3.85 | All-cause mortality |  |
|  | Q4, 2019 | 30 | 12 | 2.57 | 1.74 | 3.67 | All-cause mortality |  |
|  | Q1, 2020 | 25 | 13 | 1.87 | 1.21 | 2.77 | All-cause mortality |  |
|  | Q2, 2020 | 52 | 11 | 4.82 | 3.60 | 6.32 | All-cause mortality |  |
|  | Q3, 2020 | 20 | 10 | 2.06 | 1.26 | 3.19 | All-cause mortality |  |
|  | Q4, 2020 | 25 | 11 | 2.23 | 1.44 | 3.29 | All-cause mortality |  |
|  | Q1, 2019 | 32 | 10 | 3.14 | 2.14 | 4.43 | Non COVID19 mortality | |
|  | Q2, 2019 | 33 | 9 | 3.62 | 2.49 | 5.08 | Non COVID19 mortality | |
|  | Q3, 2019 | 26 | 9 | 2.93 | 1.92 | 4.30 | Non COVID19 mortality | |
|  | Q4, 2019 | 30 | 10 | 3.01 | 2.03 | 4.30 | Non COVID19 mortality | |
|  | Q1, 2020 | 23 | 11 | 2.05 | 1.30 | 3.08 | Non COVID19 mortality | |
|  | Q2, 2020 | 37 | 11 | 3.39 | 2.39 | 4.67 | Non COVID19 mortality | |
|  | Q3, 2020 | 21 | 8 | 2.58 | 1.59 | 3.94 | Non COVID19 mortality | |
|  | Q4, 2020 | 21 | 9 | 2.41 | 1.49 | 3.68 | Non COVID19 mortality | |
|  | Q1, 2020 | 0 | - | - | - | - | COVID-19 |  |
|  | Q2, 2020 | 14 | 6 | 2.18 | 1.19 | 3.66 | COVID-19 |  |
|  | Q3, 2020 | <10 | ND | 5.57 | 0.14 | 8.00 | COVID-19 |  |
|  | Q4, 2020 | <10 | ND | 1.46 | 0.18 | 5.28 | COVID-19 |  |
| South Asian | Q1, 2019 | 17 | 11 | 1.56 | 0.91 | 2.49 | All-cause mortality |  |
|  | Q2, 2019 | 18 | 9 | 1.99 | 1.18 | 3.15 | All-cause mortality |  |
|  | Q3, 2019 | 21 | 9 | 2.43 | 1.50 | 3.72 | All-cause mortality |  |
|  | Q4, 2019 | 22 | 10 | 2.18 | 1.37 | 3.30 | All-cause mortality |  |
|  | Q1, 2020 | 27 | 12 | 2.33 | 1.54 | 3.39 | All-cause mortality |  |
|  | Q2, 2020 | 48 | 9 | 5.17 | 3.81 | 6.86 | All-cause mortality |  |
|  | Q3, 2020 | 11 | 9 | 1.27 | 0.64 | 2.28 | All-cause mortality |  |
|  | Q4, 2020 | 23 | 10 | 2.27 | 1.44 | 3.41 | All-cause mortality |  |
|  | Q1, 2019 | 17 | 9 | 1.93 | 1.12 | 3.09 | Non COVID19 mortality | |
|  | Q2, 2019 | 18 | 8 | 2.26 | 1.34 | 3.57 | Non COVID19 mortality | |
|  | Q3, 2019 | 21 | 8 | 2.71 | 1.68 | 4.14 | Non COVID19 mortality | |
|  | Q4, 2019 | 22 | 9 | 2.54 | 1.59 | 3.85 | Non COVID19 mortality | |
|  | Q1, 2020 | 19 | 10 | 1.94 | 1.17 | 3.02 | Non COVID19 mortality | |
|  | Q2, 2020 | 25 | 9 | 2.65 | 1.71 | 3.91 | Non COVID19 mortality | |
|  | Q3, 2020 | <10 | ND | 1.37 | 0.66 | 2.52 | Non COVID19 mortality | |
|  | Q4, 2020 | 20 | 8 | 2.51 | 1.54 | 3.88 | Non COVID19 mortality | |
|  | Q1, 2020 | <10 | ND | 4.84 | 0.12 | 8.00 | COVID-19 |  |
|  | Q2, 2020 | 24 | 6 | 4.16 | 2.67 | 6.19 | COVID-19 |  |
|  | Q3, 2020 | 0 | - | - | - | - | COVID-19 |  |
|  | Q4, 2020 | <10 | ND | 1.55 | 0.19 | 5.61 | COVID-19 |  |

***Key****: ND: Not displayed; - no deaths; *All-cause mortality, age and gender standardised to five-year average weekly deaths (2015-2019) and mid-year population (2019) in England and Wales, cause-specific mortality, age and gender standardised to the general population from London (2019-2010) and mid-year population for London (2019)/ projected mid-year population from London (2020)*

**Supplementary Figure 2:** Age- and gender-standardised mortality ratios (SMRs) by psychiatric diagnoses with deaths from 2019 to end 2020, standardised to data from London*

**Legend:** Q1: quarter 1; Q2: quarter 2; Q3: quarter 3; Q4: quarter 4; a: 2019; b: 2020. SUD: Substance use disorders. Red line indicates 30^th^ January 2020, when the WHO declared COVID19 a public health emergency of international concern; *Standardised to deaths in London in 2019, and mid-year population estimates from London in 2019. Grey line indicates SMR of 1.00 (no difference compared to the standard/ reference population).

**Supplementary Figure 3: Age- and gender-standardised mortality ratios (SMRs) by ethnicity across all psychiatric diagnoses, with deaths from 2019 to end 2020, standardised to data from London***

**Legend:** Q1: quarter 1; Q2: quarter 2; Q3: quarter 3; Q4: quarter 4; a: 2019; b: 2020. Red vertical line indicates 30^th^ January 2020, when the WHO declared COVID19 a public health emergency of international concern. SMR: Standardised mortality ratio. *Standardised to deaths in London in 2019, and mid-year population estimates from London in 2019. Grey horizontal line indicates SMR of 1.00 (no difference compared to the standard/ reference population).

**Supplementary Table 3**

**Random intercepts Poisson regression; Stratum specific estimates by psychiatric diagnoses and by ethnicity, comparing deaths in quarters in 2020 to the same quarter in 2019**

|  | **Quarter 1, 2020,**  **[REF Quarter 1, 2019)** | | | | **Quarter 2, 2020,**  **[REF: Quarter 2, 2019]** | | | | **Quarter 3, 2020,**  **[REF: Quarter 3, 2019]** | | | | **Quarter 4, 2020,**  **[REF: Quarter 4. 2019]** | | | |
| --- | --- | --- | --- | --- | --- | --- | --- | --- | --- | --- | --- | --- | --- | --- | --- | --- |
|  | **IRR** | **(95% CI)** | | **p value** | **IRR** | **(95% CI)** | | **p value** | **IRR** | **(95% CI)** | | **p value** | **IRR** | **(95% CI)** | | **p value** |
| **Diagnoses** |  |  |  |  |  |  |  |  |  |  |  |  |  |  |  |  |
| Substance use disorders | 0.94 | 0.75 | 1.18 | 0.61 | **1.24** | **1.00** | **1.53** | **0.05** | 0.82 | 0.65 | 1.05 | 0.11 | 1.00 | 0.80 | 1.25 | 0.98 |
| Dementia | **1.18** | **1.03** | **1.36** | **0.02** | **2.38** | **2.08** | **2.72** | **<0.0001** | 1.04 | 0.88 | 1.23 | 0.63 | 0.97 | 0.84 | 1.12 | 0.69 |
| Schizophrenia-spectrum | 1.15 | 0.88 | 1.52 | 0.31 | **1.89** | **1.46** | **2.44** | **<0.0001** | 0.86 | 0.63 | 1.17 | 0.33 | 0.92 | 0.69 | 1.23 | 0.60 |
| Affective disorders | 1.16 | 0.97 | 1.38 | 0.10 | **1.62** | **1.38** | **1.90** | **<0.0001** | 0.86 | 0.71 | 1.05 | 0.13 | 0.93 | 0.78 | 1.11 | 0.42 |
| Neurotic/ somatoform disorders | **1.28** | **1.03** | **1.57** | **0.02** | **1.60** | **1.30** | **1.97** | **<0.0001** | 0.94 | 0.74 | 1.18 | 0.58 | 0.93 | 0.75 | 1.15 | 0.48 |
| Eating disorders | 1.01 | 0.34 | 3.01 | 0.98 | 2.91 | 0.80 | 10.56 | 0.10 | 0.73 | 0.22 | 2.39 | 0.60 | 0.58 | 0.16 | 2.04 | 0.39 |
| Pervasive developmental disorders | 0.78 | 0.30 | 2.03 | 0.62 | 1.49 | 0.68 | 3.26 | 0.32 | 0.56 | 0.22 | 1.45 | 0.23 | 1.55 | 0.52 | 4.64 | 0.43 |
| Learning disabilities | 0.72 | 0.39 | 1.32 | 0.28 | **2.67** | **1.42** | **5.03** | **<0.01** | 0.71 | 0.35 | 1.46 | 0.36 | 1.45 | 0.62 | 3.38 | 0.39 |
| Personality disorders | 1.04 | 0.70 | 1.55 | 0.85 | **1.53** | **1.04** | **2.25** | **0.03** | 0.72 | 0.45 | 1.14 | 0.16 | 1.06 | 0.68 | 1.66 | 0.79 |
| **Ethnicity** |  |  |  |  |  |  |  |  |  |  |  |  |  |  |  |  |
| White British | **1.16** | **1.03** | **1.31** | **0.02** | **1.96** | **1.73** | **2.22** | **<0.0001** | 0.97 | 0.84 | 1.12 | 0.70 | 0.97 | 0.85 | 1.10 | 0.59 |
| Black African | 1.38 | 0.80 | 2.36 | 0.25 | **1.73** | **1.03** | **2.91** | **0.04** | 1.06 | 0.55 | 2.04 | 0.86 | 1.37 | 0.75 | 2.50 | 0.30 |
| Black Caribbean | 1.27 | 0.94 | 1.71 | 0.11 | **2.48** | **1.86** | **3.30** | **<0.0001** | 0.98 | 0.71 | 1.37 | 0.92 | 1.01 | 0.74 | 1.38 | 0.94 |
| Irish | 0.75 | 0.44 | 1.26 | 0.27 | 1.52 | 0.98 | 2.35 | 0.06 | 0.79 | 0.44 | 1.41 | 0.42 | 0.87 | 0.51 | 1.47 | 0.59 |
| South Asian | 1.50 | 0.82 | 2.75 | 0.19 | **2.60** | **1.51** | **4.46** | **<0.01** | 0.52 | 0.25 | 1.09 | 0.08 | 1.04 | 0.58 | 1.87 | 0.89 |

***Key:*** *IRR: Incidence Rate Ratio; 95% CI: 95% Confidence Intervals; Stratum specific p values are from Wald tests. IRRs display ratio for observed count of deaths in each quarter in 2020 compared to the reference quarter (same quarter in 2019), taking into account the log ‘expected’ deaths as offset. Expected deaths based on age and sex distribution of the standard population. Likelihood Ratio Test (LRT) for interactions (quarters*psychiatric diagnoses) was p=0.05 and for (quarters*ethnicity) was p=0.55*

**Supplementary Table 4:** Age- and gender-standardised mortality ratios (SMRs) across

psychiatric diagnoses, by cause (COVID-19 mortality and deaths from all other/

non-COVID-19 related causes) by quarters in 2019 and 2020.

| **Diagnosis** | **Quarter, year** | **Observed deaths** | **Expected deaths** | **SMR** | **(95% CI)** | | **Cause of death** | |
| --- | --- | --- | --- | --- | --- | --- | --- | --- |
| Substance use disorders (F1*) | Q1, 2019 | 76 | 28 | 2.71 | 2.14 | 3.40 | All other causes | |
|  | Q2, 2019 | 102 | 26 | 3.86 | 3.15 | 4.69 | All other causes | |
|  | Q3, 2019 | 117 | 27 | 4.39 | 3.63 | 5.27 | All other causes | |
|  | Q4, 2019 | 128 | 28 | 4.55 | 3.80 | 5.41 | All other causes | |
|  | Q1, 2020 | 138 | 32 | 4.38 | 3.68 | 5.17 | All other causes | |
|  | Q2, 2020 | 162 | 32 | 5.09 | 4.34 | 5.94 | All other causes | |
|  | Q3, 2020 | 108 | 29 | 3.71 | 3.04 | 4.48 | All other causes | |
|  | Q4, 2020 | 124 | 31 | 4.04 | 3.36 | 4.82 | All other causes | |
|  | Q1, 2020 | 0 | - | - | - | - | COVID19 |  |
|  | Q2, 2020 | 38 | 22 | 1.76 | 1.25 | 2.42 | COVID19 |  |
|  | Q3, 2020 | <10 | ND | 3.48 | 0.42 | 12.56 | COVID19 |  |
|  | Q4, 2020 | <10 | ND | 0.74 | 0.15 | 2.17 | COVID19 |  |
| Dementia (F0*) | Q1, 2019 | 340 | 149 | 2.28 | 2.04 | 2.54 | All other causes | |
|  | Q2, 2019 | 299 | 133 | 2.24 | 2.00 | 2.51 | All other causes | |
|  | Q3, 2019 | 273 | 127 | 2.15 | 1.90 | 2.42 | All other causes | |
|  | Q4, 2019 | 366 | 148 | 2.47 | 2.22 | 2.74 | All other causes | |
|  | Q1, 2020 | 397 | 165 | 2.41 | 2.17 | 2.65 | All other causes | |
|  | Q2, 2020 | 457 | 158 | 2.89 | 2.64 | 3.17 | All other causes | |
|  | Q3, 2020 | 249 | 110 | 2.27 | 2.00 | 2.57 | All other causes | |
|  | Q4, 2020 | 306 | 121 | 2.54 | 2.26 | 2.84 | All other causes | |
|  | Q1, 2020 | <10 | ND | 2.76 | 1.26 | 5.24 | COVID19 |  |
|  | Q2, 2020 | 335 | 88 | 3.82 | 3.42 | 4.25 | COVID19 |  |
|  | Q3, 2020 | <10 | ND | 3.62 | 1.66 | 6.88 | COVID19 |  |
|  | Q4, 2020 | 30 | 20 | 1.50 | 1.02 | 2.15 | COVID19 |  |
| Schizophrenia-spectrum disorders (F2*) | Q1, 2019 | 60 | 33 | 1.83 | 1.40 | 2.36 | All other causes | |
|  | Q2, 2019 | 65 | 30 | 2.16 | 1.66 | 2.75 | All other causes | |
|  | Q3, 2019 | 62 | 30 | 2.08 | 1.60 | 2.67 | All other causes | |
|  | Q4, 2019 | 86 | 33 | 2.63 | 2.10 | 3.25 | All other causes | |
|  | Q1, 2020 | 90 | 37 | 2.46 | 1.98 | 3.03 | All other causes | |
|  | Q2, 2020 | 97 | 36 | 2.69 | 2.18 | 3.28 | All other causes | |
|  | Q3, 2020 | 65 | 30 | 2.16 | 1.67 | 2.76 | All other causes | |
|  | Q4, 2020 | 72 | 32 | 2.25 | 1.76 | 2.83 | All other causes | |
|  | Q1, 2020 | <10 | ND | 2.82 | 0.34 | 10.19 | COVID19 |  |
|  | Q2, 2020 | 73 | 22 | 3.26 | 2.55 | 4.10 | COVID19 |  |
|  | Q3, 2020 | <10 | ND | 1.61 | 0.04 | 8.96 | COVID19 |  |
|  | Q4, 2020 | <10 | ND | 1.73 | 0.75 | 3.41 | COVID19 |  |
| Affective disorders (F3*) | Q1, 2019 | 168 | 83 | 2.01 | 1.72 | 2.34 | All other causes | |
|  | Q2, 2019 | 192 | 77 | 2.51 | 2.17 | 2.89 | All other causes | |
|  | Q3, 2019 | 198 | 75 | 2.64 | 2.28 | 3.03 | All other causes | |
|  | Q4, 2019 | 200 | 83 | 2.41 | 2.08 | 2.76 | All other causes | |
|  | Q1, 2020 | 250 | 93 | 2.68 | 2.36 | 3.04 | All other causes | |
|  | Q2, 2020 | 245 | 91 | 2.70 | 2.37 | 3.06 | All other causes | |
|  | Q3, 2020 | 176 | 74 | 2.39 | 2.05 | 2.77 | All other causes | |
|  | Q4, 2020 | 193 | 79 | 2.44 | 2.11 | 2.82 | All other causes | |
|  | Q1, 2020 | <10 | ND | 3.35 | 1.23 | 7.29 | COVID19 |  |
|  | Q2, 2020 | 164 | 55 | 2.97 | 2.53 | 3.46 | COVID19 |  |
|  | Q3, 2020 | <10 | ND | 2.56 | 0.70 | 6.54 | COVID19 |  |
|  | Q4, 2020 | 13 | 12 | 1.13 | 0.60 | 1.93 | COVID19 |  |
| Neurotic, stress related and somatoform disorders (F4*) | Q1, 2019 | 112 | 57 | 1.97 | 1.62 | 2.37 | All other causes | |
|  | Q2, 2019 | 117 | 53 | 2.22 | 1.84 | 2.66 | All other causes | |
|  | Q3, 2019 | 111 | 52 | 2.14 | 1.76 | 2.57 | All other causes | |
|  | Q4, 2019 | 155 | 58 | 2.66 | 2.26 | 3.12 | All other causes | |
|  | Q1, 2020 | 177 | 65 | 2.71 | 2.33 | 3.14 | All other causes | |
|  | Q2, 2020 | 175 | 64 | 2.74 | 2.35 | 3.18 | All other causes | |
|  | Q3, 2020 | 122 | 53 | 2.32 | 1.92 | 2.77 | All other causes | |
|  | Q4, 2020 | 132 | 57 | 2.33 | 1.95 | 2.76 | All other causes | |
|  | Q1, 2020 | <10 | ND | 2.43 | 0.50 | 7.10 | COVID19 |  |
|  | Q2, 2020 | 92 | 38 | 2.39 | 1.93 | 2.94 | COVID19 |  |
|  | Q3, 2020 | <10 | ND | 2.72 | 0.56 | 7.96 | COVID19 |  |
|  | Q4, 2020 | <10 | ND | 1.23 | 0.59 | 2.26 | COVID19 |  |
| Eating disorders (F50) | Q1, 2019 | <10 | ND | 1.22 | 0.15 | 4.41 | All other causes | |
|  | Q2, 2019 | <10 | ND | 1.83 | 0.38 | 5.35 | All other causes | |
|  | Q3, 2019 | <10 | ND | 4.42 | 1.78 | 9.10 | All other causes | |
|  | Q4, 2019 | <10 | ND | 2.24 | 0.61 | 5.72 | All other causes | |
|  | Q1, 2020 | <10 | ND | 1.07 | 0.13 | 3.86 | All other causes | |
|  | Q2, 2020 | <10 | ND | 3.17 | 1.16 | 6.91 | All other causes | |
|  | Q3, 2020 | <10 | ND | 3.30 | 1.21 | 7.18 | All other causes | |
|  | Q4, 2020 | <10 | ND | 1.02 | 0.12 | 3.67 | All other causes | |
|  | Q1, 2020 | 0 | - | - | - | - | COVID19 |  |
|  | Q2, 2020 | <10 | ND | 4.81 | 1.56 | 11.22 | COVID19 |  |
|  | Q3, 2020 | 0 | - | - | - | - | COVID19 |  |
|  | Q4, 2020 | 0 | - | - | - | - | COVID19 |  |
| Pervasive developmental disorders (F8*) | Q1, 2019 | <10 | ND | 0.69 | 0.08 | 2.48 | All other causes | |
|  | Q2, 2019 | <10 | ND | 2.48 | 1.00 | 5.10 | All other causes | |
|  | Q3, 2019 | 13 | 3 | 4.44 | 2.36 | 7.59 | All other causes | |
|  | Q4, 2019 | <10 | ND | 1.59 | 0.51 | 3.70 | All other causes | |
|  | Q1, 2020 | <10 | ND | 1.47 | 0.48 | 3.43 | All other causes | |
|  | Q2, 2020 | <10 | ND | 3.11 | 1.49 | 5.71 | All other causes | |
|  | Q3, 2020 | <10 | ND | 1.24 | 0.34 | 3.17 | All other causes | |
|  | Q4, 2020 | <10 | ND | 1.16 | 0.32 | 2.98 | All other causes | |
|  | Q1, 2020 | 0 | - | - | - | - | COVID19 |  |
|  | Q2, 2020 | <10 | ND | 5.01 | 2.40 | 9.20 | COVID19 |  |
|  | Q3, 2020 | 0 | - | - | - | - | COVID19 |  |
|  | Q4, 2020 | 0 | - | - | - | - | COVID19 |  |
| Learning disabilities (F7*) | Q1, 2019 | 18 | 4 | 4.46 | 2.64 | 7.05 | All other causes | |
|  | Q2, 2019 | 15 | 4 | 4.11 | 2.30 | 6.79 | All other causes | |
|  | Q3, 2019 | 15 | 4 | 4.10 | 2.29 | 6.76 | All other causes | |
|  | Q4, 2019 | 11 | 4 | 2.77 | 1.38 | 4.95 | All other causes | |
|  | Q1, 2020 | 13 | 4 | 2.98 | 1.59 | 5.09 | All other causes | |
|  | Q2, 2020 | 16 | 4 | 3.79 | 2.17 | 6.16 | All other causes | |
|  | Q3, 2020 | <10 | ND | 2.21 | 0.95 | 4.36 | All other causes | |
|  | Q4, 2020 | 14 | 4 | 3.65 | 1.99 | 6.12 | All other causes | |
|  | Q1, 2020 | 0 | - | - | - | - | COVID19 |  |
|  | Q2, 2020 | 25 | 3 | 9.24 | 5.98 | 13.64 | COVID19 |  |
|  | Q3, 2020 | <10 | ND | 13.76 | 0.35 | 76.68 | COVID19 |  |
|  | Q4, 2020 | 0 | - | - | - | - | COVID19 |  |
| Personality disorders (F6*) | Q1, 2019 | 20 | 9 | 2.23 | 1.36 | 3.44 | All other causes | |
|  | Q2, 2019 | 25 | 9 | 2.92 | 1.89 | 4.32 | All other causes | |
|  | Q3, 2019 | 34 | 8 | 4.02 | 2.78 | 5.61 | All other causes | |
|  | Q4, 2019 | 30 | 9 | 3.24 | 2.19 | 4.63 | All other causes | |
|  | Q1, 2020 | 43 | 10 | 4.19 | 3.03 | 5.64 | All other causes | |
|  | Q2, 2020 | 28 | 10 | 2.74 | 1.82 | 3.96 | All other causes | |
|  | Q3, 2020 | 23 | 9 | 2.52 | 1.60 | 3.78 | All other causes | |
|  | Q4, 2020 | 33 | 10 | 3.40 | 2.34 | 4.77 | All other causes | |
|  | Q1, 2020 | 0 | - | - | - | - | COVID19 |  |
|  | Q2, 2020 | 30 | 7 | 4.58 | 3.09 | 6.53 | COVID19 |  |
|  | Q3, 2020 | <10 | ND | 5.41 | 0.14 | 30.14 | COVID19 |  |
|  | Q4, 2020 | <10 | ND | 2.40 | 0.49 | 7.01 | COVID19 |  |

***Key****: ND: Not displayed; - no deaths; Age and gender standardised to the general population from London (2019-2010) and mid-year population for London (2019)/ projected mid-year population from London (2020)*

**Supplementary Figure 4:**

Sensitivity analysis: Comparison of age and gender SMRs across time in Affective disorders (ICD-10 code F3*) with other psychiatric diagnoses (‘comorbidities’) versus no other psychiatric diagnoses (‘no comorbidities’)

***Key:*** *Standardised to five-year average weekly deaths (2015-2019) and mid-year population (2019) in England and Wales. Q1: quarter 1; Q2: quarter 2; Q3: quarter 3; Q4: quarter 4; Vertical red line indicates 30^th^ January 2020, when the WHO declared COVID-19 a public health emergency of international concern; Grey line indicates SMR of 1.00 (no difference compared to the standard/ reference population).* *SMR: Standardised mortality ratio.*
